# Domestication Syndrome Is Investigated by Proteomic Analysis between Cultivated Cassava (*Manihot esculenta* Crantz) and Its Wild Relatives

**DOI:** 10.1371/journal.pone.0152154

**Published:** 2016-03-29

**Authors:** Feifei An, Ting Chen, Djabou Mouafi Astride Stéphanie, Kaimian Li, Qing X. Li, Luiz J. C. B. Carvalho, Keith Tomlins, Jun Li, Bi Gu, Songbi Chen

**Affiliations:** 1 Tropical Crops Genetic Resources Institute, Chinese Academy of Tropical Agricultural Sciences/Key Laboratory of Ministry of Agriculture for Germplasm Resources Conservation and Utilization of Cassava, Danzhou 571737, China; 2 Laboratory of Plant Physiology, Higher Teacher’s Training College, University of Yaounde I, P. O. Box 47, Yaounde, Cameroon; 3 Department of Molecular Biosciences and Bioengineering, University of Hawaii at Manoa, Manoa, HI 96822, United States of America; 4 Genetic Resources and Biotechnology, Embrapa, Brasilia-DF 02372, Brazil; 5 Natural Resources Institute, University of Greenwich, Chatham ME4 4TB, United Kingdom; 6 Analysis and Testing Center, Jiangsu University, Zhenjiang 212013, China; 7 Chemical Starch Institute, Guangxi University, Nanning 300004, China; Shanghai Institutes for Biological Sciences, CHINA

## Abstract

Cassava (*Manihot esculenta* Crantz) wild relatives remain a largely untapped potential for genetic improvement. However, the domestication syndrome phenomena from wild species to cultivated cassava remain poorly understood. The analysis of leaf anatomy and photosynthetic activity showed significantly different between cassava cultivars SC205, SC8 and wild relative *M*. *esculenta* ssp. *Flabellifolia* (W14). The dry matter, starch and amylose contents in the storage roots of cassava cultivars were significantly more than that in wild species. In order to further reveal the differences in photosynthesis and starch accumulation of cultivars and wild species, the globally differential proteins between cassava SC205, SC8 and W14 were analyzed using 2-DE in combination with MALDI-TOF tandem mass spectrometry. A total of 175 and 304 proteins in leaves and storage roots were identified, respectively. Of these, 122 and 127 common proteins in leaves and storage roots were detected in SC205, SC8 and W14, respectively. There were 11, 2 and 2 unique proteins in leaves, as well as 58, 9 and 12 unique proteins in storage roots for W14, SC205 and SC8, respectively, indicating proteomic changes in leaves and storage roots between cultivated cassava and its wild relatives. These proteins and their differential regulation across plants of contrasting leaf morphology, leaf anatomy pattern and photosynthetic related parameters and starch content could contribute to the footprinting of cassava domestication syndrome. We conclude that these global protein data would be of great value to detect the key gene groups related to cassava selection in the domestication syndrome phenomena.

## Introduction

Cassava (*Manihot esculenta* Crantz) is the world’s most important non-grain food crop which provides global food security and income generation throughout tropical Africa, Asia, and the Americas for its starchy storage roots [1]. The advantages of cassava over other crops are high productivity and adaptability to various stress condition, thus it is farmer favorable. Cassava originated in South America was domesticated to Africa less than 10,000 years ago by European sailor and then traders introduce the plant to Asia. [2]. As a result, cassava is now the most important dietary source of calories in the tropics after rice and maize and feed an estimated 800 million people throughout the world [3, 4]. Despite its importance, the nutritional value of cassava is limited as the roots contain little protein [5] and high levels of cyanogenic compounds [6]. In addition, postharvest deterioration is rapidly happened after wounding, leading to shorten shelf-life and limiting economy development [7]. Cassava is a heterozygous nature species with a high genetic load which presents difficulties in the identification of the parents with good breeding values due to generation of new segregating progenies [8]. Together, these properties present a significant barrier to the already slow process of improving yield, reducing postharvest deterioration and increasing nutrient content using classical breeding approaches [9]. A challenge to the scientific community is to obtain a genome sequence that will facilitate improved breeding.

Wild cassava species are untapped resources for the genetic enhancement of cassava. Selection through domestication has resulted in many morphological, physiological and biochemical differences between cassava and its wild ancestor. Some traits, such as increased size of the root and higher starch content and vegetative propagation through stem cuttings are the result of human selection [10, 11]. To overcome the key issue of postharvest deterioration and other limitations to generate a higher-quality of cassava cultivars, the hybridization of cassava with its closely wild relatives has been performed. Wild cassava possesses useful genes that if incorporated into the cultigen would enrich its gene pool with useful characters related to its consumption or adaptation to more severe conditions of soil and climate. Systematic interspecific hybridization was undertaken to broaden its genetic base with genes of the wild species [12]. *M*. *esculenta* subsp. *Flabellifolia* (W14) is regarded as the wild progenitor of modern cultivars and thus part of the primary gene pool of the root crop [13]. The more closely related the wild species is to cultivated cassava, the more successful hybridization seems to become; for example, 16 successful crosses at CIAT between cassava and the conspecific wild progenitor W14 resulted in “thousands of seeds’, whereas only five seeds of unknown viability were obtained from two crosses with *M*. *aesculifolia* [14]. Wild cassava can also provide genes for low cyanide content and for African cassava mosaic diseases (CMD) resistance. For some other characteristics, such as resistance to cassava bacterial blight (CBB) or high starch content, certain sources of genes have been identified [15]. The hybrids of *M*. *esculenta* with its wild relatives, *M*. *oligantha* were shown to significantly increase crude protein content and essential amino acids, and decrease the levels of total cyanide [2]. It is reported from CIAT that the F1 generations crossed from W14 and *M*. *esculenta* were used to hybridize with *M*. *tristisand* and W14 to generate high protein content cassava, as well as hybridize with *M*. *walkierae* to generate reduced post-harvest physiological deterioration cassava. The combined data resources allowed us to explore wild cassava potential for improvement of cassava yield and nutrition.

Cassava whole genome sequence and many expressed sequence tags are now publicly available. These resources will accelerate marker-assisted breeding, allowing improvements in disease-resistance and nutrition, and it will be helpful to understand the genetic basis for disease resistance [9]. Cassava online archive database is available at http://cassava.psc.riken.jp/, allowing searches with gene function, accession number, and sequence similarity (BLAST) [16]. Although cassava genome sequence is an information resource, the value of the genome is its annotation, which bridges the gap from the sequence to the biology of cassava. Cassava genome is a multi-step process, including three categories: nucleotide-level, protein-level and process-level annotation [17]. Despite the recently significant advances on the nucleotide-level annotation, very little is known about the cassava global protein-level annotation, particularly focusing on wild species existing in the world.

Proteomics is a useful tool to compile a definitive catalogue of cassava global proteins, to name them and to assign them putative functions, providing a global protein-level annotation for cassava whole genome. It is applied to all protein expression in a particular organelle or tissue or in response to a particular stress. Proteomic analysis has revealed which proteins are responsible for cell differentiation in *Arabidopsis* under salt and osmotic stress and drought responsiveness in maritime pine, maize and wild watermelon [18]. In cassava, proteomics was employed to compare proteome patterns between fibrous and storage roots [19] and also used to describe the proteome characteristics of somatic embryos, plantlets and storage roots in cassava SC8 [8]. Owiti *et al*. (2011) investigated the molecular changes during physiological deterioration of cassava root after harvest using isobaric tags for relative and absolute quantification of proteins in soluble and non-soluble fractions prepared during a 96 h post-harvest time course, establishing a comprehensive proteome map of the cassava root and identified quantitatively regulated proteins [7]. Recently, An *et al*. (2014) employed a proteomic method to detect the changes of cassava polyploidy genotypes at proteome levels, and provided an insight into understanding the protein regulation mechanism of cassava polyploidy genotype [6]. However, the proteome diversity between cassava cultivars and its wild relatives is poorly understood.

The purpose of the present study was to compare the differences of anatomy, physiology and proteomes in leaves and storage roots between cassava cultivars and wild relative W14. All identified proteins were classified into cohesive groups based on their biochemical functions and indicated proteome diversity. The biological network of protein-protein interaction was set up to describe differential proteins regulations in the photosynthesis and starch accumulation. The proteome differences were supported by cassava anatomic and physiological data. This study will provide important clues on the improvement of cassava breeding through exploring the key gene groups related to the domestication syndrome phenomena.

## Materials and Methods

### Plant materials

Two cassava cultivars, *M*. *esculenta* cv. SC205 and SC8, and cassava’s closest wild relative *M*. *esculenta* ssp. *Flabellifolia* (W14) were selected for the present study. SC205 and SC8 were released from Tropical Crops Genetic Resources Institute (TCGRI), CATAS. W14 originated in Brazil and is currently planted in Cassava Germplasm Bank (CGB), TCGRI, CATAS. The stem cuttings of SC205, SC8 and W14 were grown in the field at CGB on February 2012. The functional leaves of SC205, SC8 and W14 grown for three months and storage roots grown for ten months were taken. Three replicates consisting of three leaf/root slices each were sampled and immediately used for microscopy observation, and also frozen in liquid nitrogen for protein extraction.

### Morphological observation under light microscopy and scanning electron microscopy

Morphological observation under light microscopy of SC205, SC8 and W14 was conducted as previously described in An *et al*. (2014) [6]. Structural changes of cassava starch granules, extracted from storage roots between SC205, SC8 and W14, were observed under scanning electron microscopy (SEM). The samples (dried starch powder) were mounted on SEM stubs with double-sided adhesive tape and coated with gold. Scanning electron micrographs were taken using an S-3400N scanning microscope (Hitachi) in Jiangsu University, China [20].

### Photosynthetic activity measurement by imaging pulse amplitude modulation

The Maxi-version of the Imaging Pulse Amplitude Modulation (Imaging PAM) and the software Imaging WIN version 2.39 (both Heinz Walz GmbH, Effeltrich, Germany) were used to determine the photosynthetic activities of W14, SC205 and SC8 according to An *et al*. (2014). For each genotype, three individual plants were used and the results were averaged.

### Determination of dry matter content, starch content and starch component

Dry matter content (DMC), starch content, and starch component including Amylose contents (AC) and amylopectin contents (APC) were measured as previously described by Gu *et al*. (2013) [21].

### Protein extraction, 2-DE separation and identification

Proteins from functional leaves and storage roots of SC205, SC8 and W14 were extracted with phenol extraction according to Chen *et al*. (2009) [18]. Protein separation was conducted following the previous described in An et al. (2014) [6]. Three independent biological replications were carried out. Gel matching for protein quantification was performed using an Image Scanner III (GE healthcare) and Delta 2D (Decodon GmbH, Greifswald, Germany) software, and spot pairs were confirmed visually. The significance of differences was determined by Scheffe’s test at P <0.05. The abundance of each protein spot was estimated by the percentage volume (%Vol). Tryptic in-gel digestion and Protein identification were performed by the methods reported in An *et al*. (2014) [6].

### Western blot analyses

Proteins of functional leaves and storage roots of three cassava genotypes were extracted [8]. Western blotting was performed according to the method previously reported [6]. Proteins detected by immuno-staining with anti-Rubisco-polyclonal antibody (AS07218), anti-OEC antibody (AS 05092) and anti-D1 antibody (AS05084) from Agrisera for leaves, anti-GBSS1 antibody and anti-linamarase antibody, produced by GenScript, anti-beta-amylase antibody (AS09379) from Agrisera for storage roots. Western blots were developed according to the method of NBT/BCIP from Roche (11681451001).

### Generation of protein interaction networks

Nineteen differential proteins involved in photosynthesis and 11 differential proteins related with starch accumulation were identified from leaves and storage roots of W14, SC205 and SC8 respectively, were used to generate the wider protein interaction maps by employing a Pathway Studio software program (www.Ariadnegenomics.com) [6].

## Results

### Plant morphology, leaf anatomy and photosynthetic capacity between cultivars and wild relatives

S1 Fig shows morphological characteristics of W14, SC205 and SC8 plants. The plant height of W14 was approximately 3.0–4.0 m (S1a1 Fig), SC205 about 2.0–2.5 m (S1b1 Fig) and SC8 about 2.0–2.5 m (S1c1 Fig) [20]. Shapes of central lobes of W14, SC205 and SC8 were lanceolate (S1a2 Fig), linear (S1b2 Fig) and elliptic (S1c2 Fig) respectively. W14 and SC8 storage roots had white flesh and white yellow skin, while SC205 had white flesh and brown skin (S1a3–S1c3 Fig).

**Fig 1:**
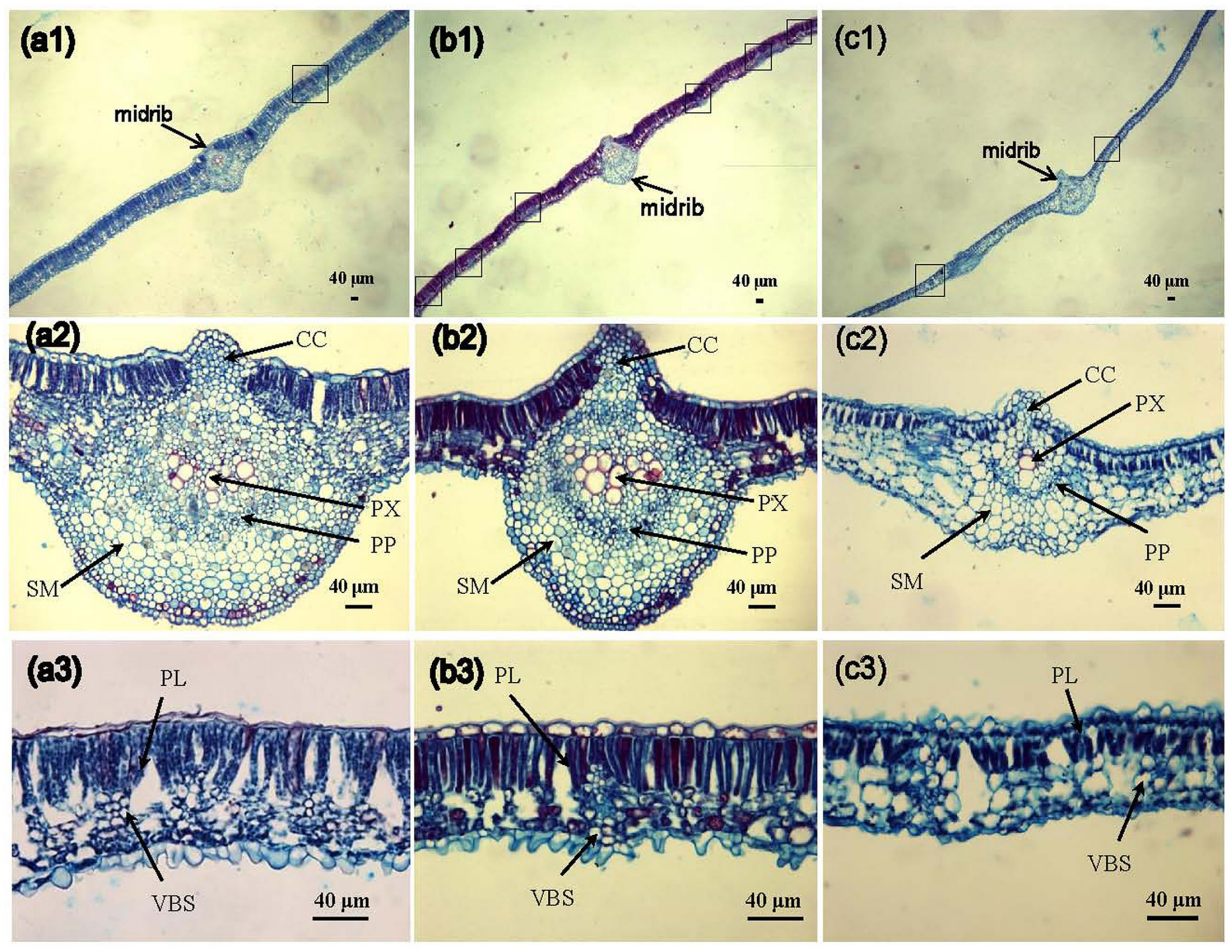
Photomicrographs of leaf transverse sections of cassava cultivars and wild relatives. (a1), (b1) and (c1), leave midrib of W14, SC205 and SC8, respectively; (a2), (b2) and (c2), Amplification of leave midrib of W14, SC205 and SC8, respectively; Primary xylem (PX); Primary Phloem (PP); Spongy mesophyll (SM); Collenchyma (CC); (a3), (b3) and (c3), leaf transverse sections of W14, SC205 and SC8, respectively. Note the long single palisade layer (PL) and the conspicuous green vascular bundle sheath (VBS) cells situated beneath the palisade layer. Scale bar = 40 μm.

Fig 1 shows leaf transverse sections of two cassava cultivars SC205 and SC8 versus the wild relative W14. The measurements of all leaf strata, including midrib, cuticle, epidermal and mesophyll layers, revealed significant differences between the cultivars and the wild relative. The most noticeable difference between the species existed in the midrib (Fig 1a1, 1b1 and 1c1). Amplification of midrib showed that SC8 had a small area of primary xylem (PX), primary phloem (PP) and collenchyma (CC) less than those of SC205 and wild relative (Fig 1a2, 1b2 and 1c2). Compared to the wild relative, the cassava cultivars had a more distinctive bundle sheath with small and thin-walled cells. In the cultivars, the vascular bundles occur below the layers of elongated palisade cells. While part of the bundle sheath was in contact with palisade cells, there was not a uniform layer of mesophyll cells around the bundle sheath as it found in C_4_ plants (Fig 1a3, 1b3 and 1c3).

**Fig 2:**
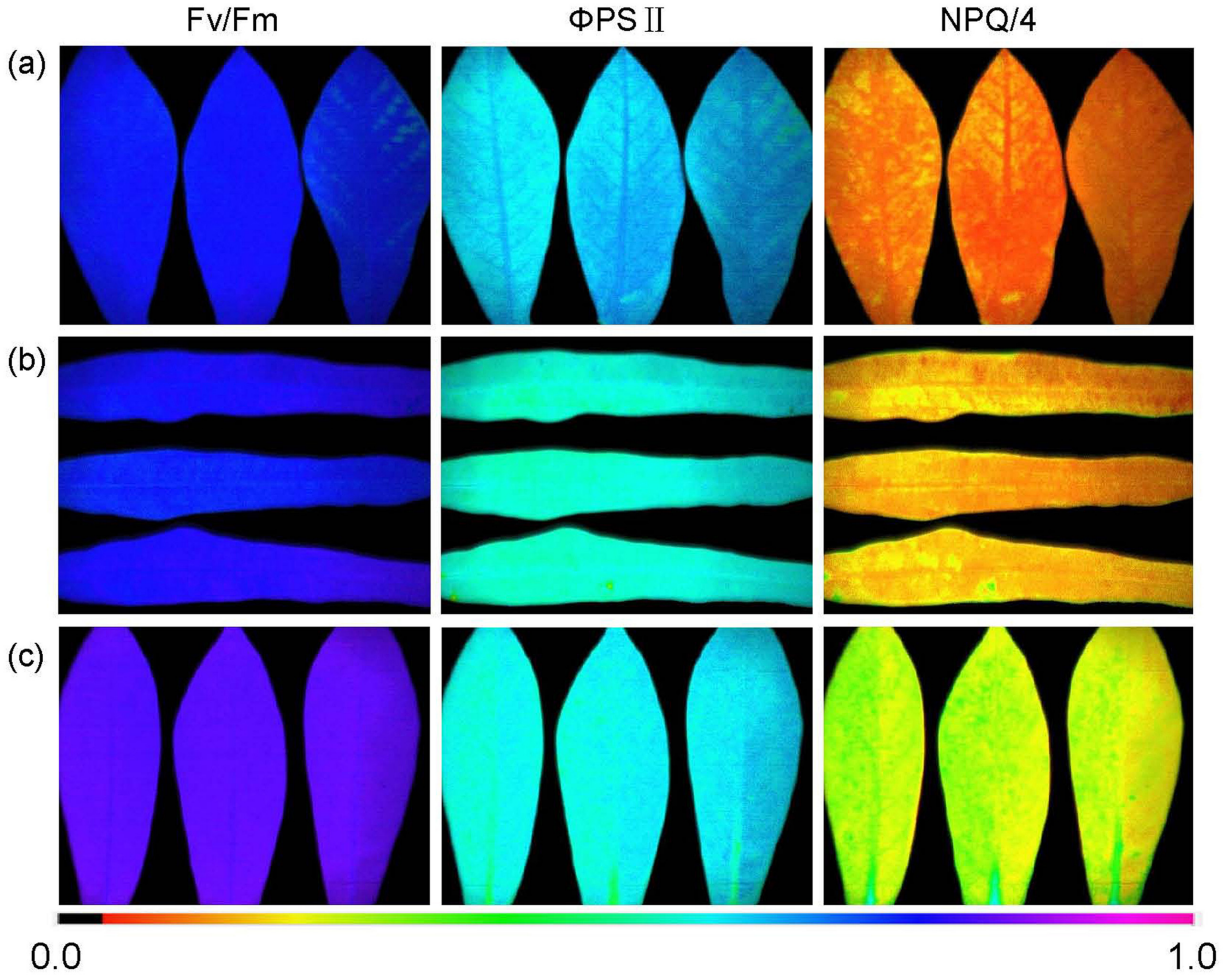
Imaging pulse amplitude modulation of W14 (a), SC205 (b) and SC8 (c) leaves. Parameters were Fv/Fm [maximal photosystem II (PSII) quantum yield], ΦPSII (effective PSII quantum yield) (at 185μE m^-2^ s^-1^), and NPQ/4 (nonphotochemical quenching) (at 185μE m^-2^s^-1^). The color gradient provided a scale from 0 to 100% for assessing the magnitude of the parameters.

In the leaves of 3 month-old cassava plants SC205, SC8 and W14, the photosynthetic abilities of SC205 and SC8 were greater than that of W14 (Fig 2 and Table 1), indicating variations in the efficiency of excitation energy capture by open Fv/Fm, *ΦPSII* and NPQ/4. These data imply that an increase in maximal and effective quantum yield and a concomitant increase in NPQ/4 processes are sensitive markers for cassava genotypes.

**Table 1:** Photosynthetic parameters collected from cassava leaves of W14, SC205 and SC8. Values were means **±** SE. Different capital letters in the same column indicated statistically significant differences according to Duncan test (P<0.01).

Cassava varieties	Fv/Fm (Mean±SE)	ΦPSII (Mean±SE)	NPQ/4 (Mean±SE)
W14	0.742±0.015 C	0.539±0.016 C	0.114±0.008 C
SC205	0.786±0.024 B	0.566±0.012 B	0.142±0.010 B
SC8	0.818±0.026 A	0.582±0.021 A	0.163±0.013 A

### Analysis of starch properties from storage roots

**Fig 3:**
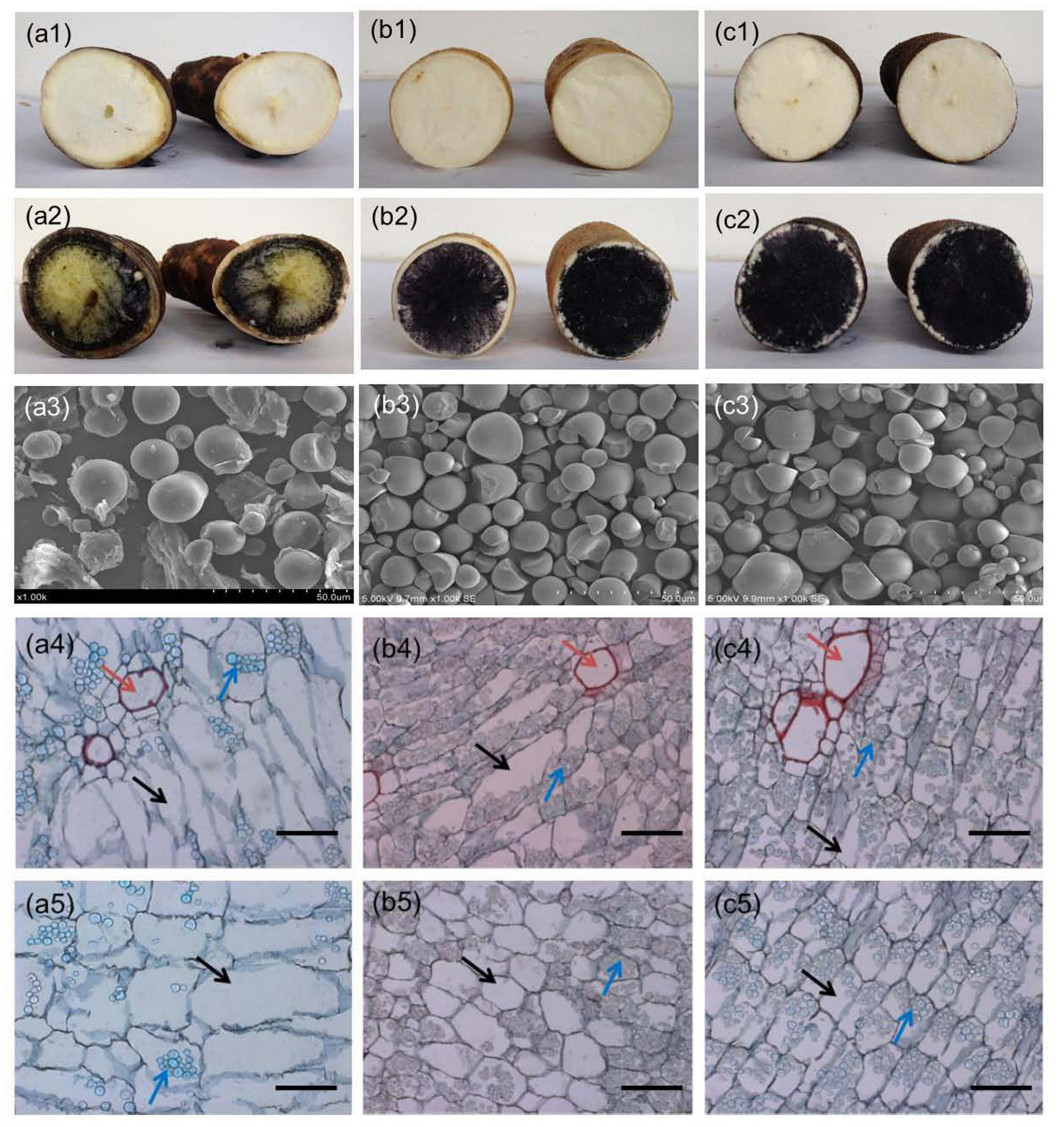
Starch staining with KI in storage roots and SEM pictures of starch granules incubated with cell-free supernatants, and paraffin section of transverse and longitude of storage roots of SC205, SC8 and W14. Paraffin section of transverse and longitude of storage roots of SC205, SC8 and W14, stained with Safranin O/Fast green and viewed under light microscope X20. (a1), (b1) and (c1), transverse sections of cassava genotype W14, SC205 and SC8, respectively; (a2), (b2) and (c2), starch staining with KI of cassava genotype W14, SC205 and SC8, respectively; (a3), (b3) and (c3), SEM of starch granules of cassava W14, SC205 and SC8, respectively. SEM magnification time was 1000; scale bar = 50 μm. (a4)-(c4), transverse sections of storage roots from W14, SC205 and SC8; (a5)-(c5), longitude sections of storage roots from W14, SC205and SC8. Scale bar = 100 μm. It shows more vessel grouping and tyloses with starch granules. SC205 and SC8 showing parenchyma cells with more starch granules, while W14 showing parenchyma cells with a little starch, these cells are bigger. Red arrows indicate xylem vessel, black arrow shows parenchyma cell, and blue arrow presents starch granules.

The highest dry matter content (DMC) of storage root was cultivar SC205 with a mean of 77.08%, reversely; the lowest DMC was wild relatives W14 with an average of 57.80% (Table 2). Cultivars SC205 and SC8 with starch contents (28.86% and 29.74%, respectively) were at least seven times more than wild relative W14 with 3.75%. Amylose contents (ACs) in SC205 and SC8 (18.81% and 19.15%, respective) were significantly greater than that of W14 (6.72%), whereas amylopectin contents (APCs) in cultivars were significantly less than wild relative (Table 2). The results regarding starch staining with KI also supported the described above (Fig 3a1–3c1 and 3a2–3c2). No significant differences of DMC, starch content, AC and APC were observed in SC205 and SC8, however, there was a significantly difference between the cultivars and the wild relative. Granule size and shape varied largely between cultivars and wild relative (Fig 3a3–3c3). No significant differences were observed in the starch granules in SC205 and SC8. The transverse (Fig 3a4–3c4) and longitude (Fig 3a5–3c5) sections of storage roots showed greater number of starch granules in SC205 and SC8 than that in the wild relative W14.

**Table 2:** Dry matter content, starch content, amylose and amylopectin content in storage roots of W14, SC205 and SC8. Values were means **±** SE. Different capital letters in the same column indicated statistically significant differences according to Duncan test(P<0.01).

Cassava Varieties	Dry Matter Content (%) (Mean±SE)	Starch Content (%) (Mean±SE)	Amylose Content (%) (Mean±SE)	Amylopectin Content (%) (Mean±SE)
W14	57.80±0.14 B	3.75±0.01 B	6.72±0.02 B	93.28±0.02 A
SC205	77.08±1.21 A	28.86±1.12 A	18.81±0.30 A	81.19±0.30 B
SC8	75.76±2.45 A	29.74±1.07 A	19.15±1.51 A	80.85±1.51 B

### Leaf protein profiles

**Fig 4:**
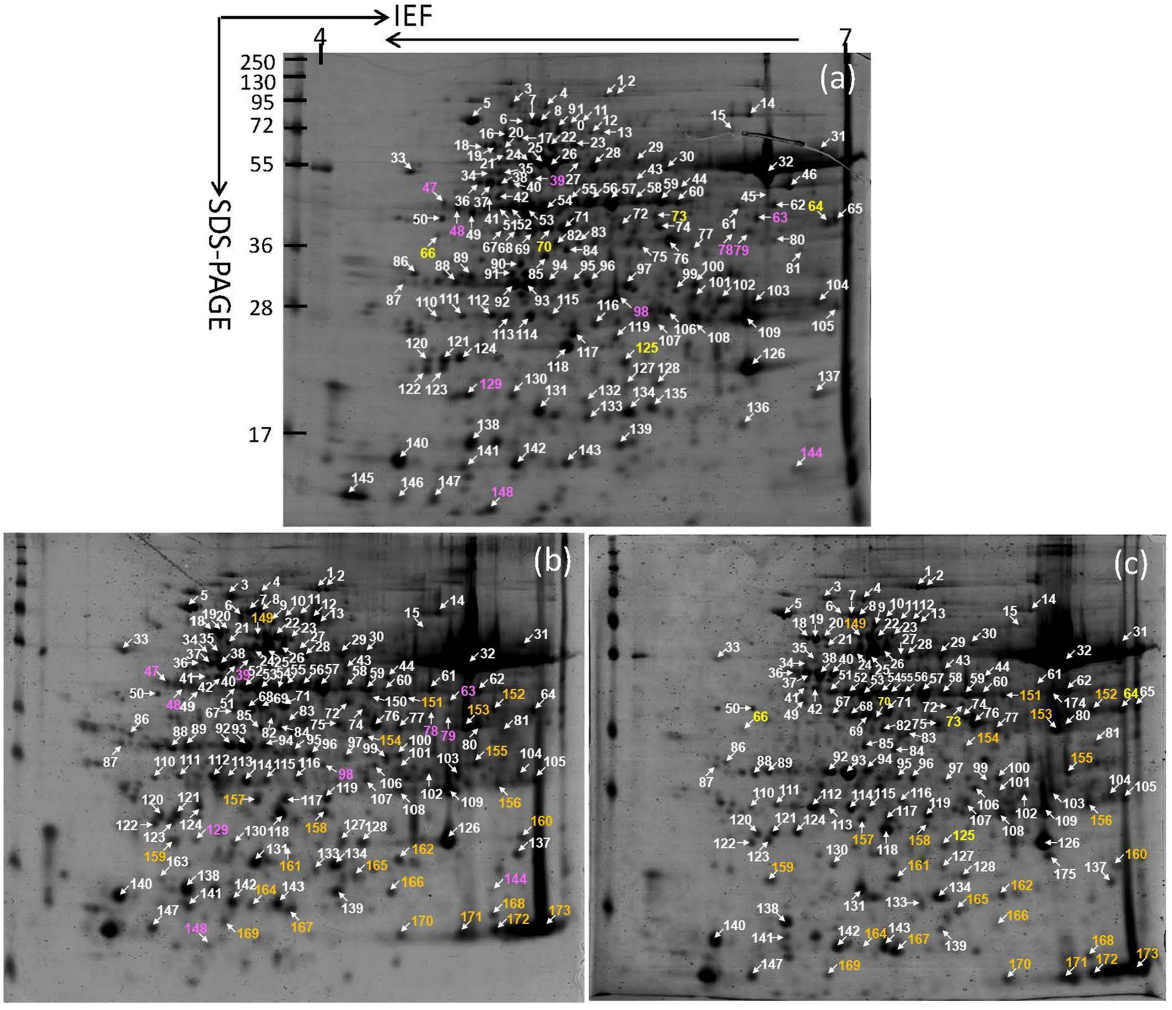
148, 157 and 152 proteins identified by MALDI-TOF-TOF-MS/MS in 2-D gel protein profiles of W14(a), SC205(b) and SC8(c) leaves, respectively. The pink numbers are common proteins to W14 and SC205, the yellow numbers are common proteins to W14 and SC8, and the orange numbers are common proteins to SC205 and SC8.

**Fig 5:**
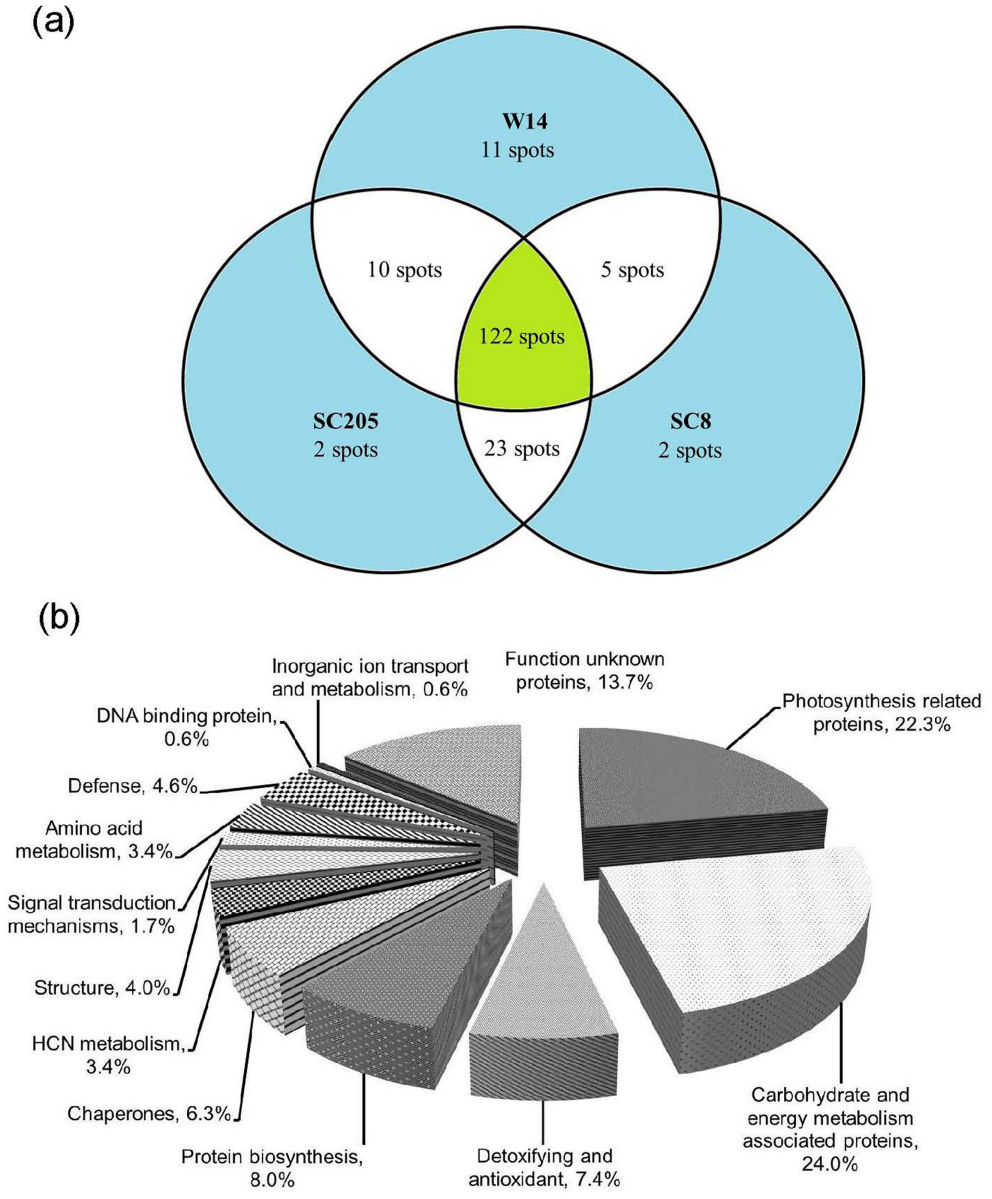
Venn diagrams of 175 proteins identified (a) and their functional classification (b) in leaves of SC205, SC8 and W14. Functional categorization was performed according to the MIPS database (http://mips.gsf.de).

Fig 4 shows 2-D gel images of W14, SC205 and SC8 leaves respectively. More than 300 protein spots on the image of each cassava genotype were analyzed, among which 148, 157 and 152 protein spots were identified in W14 (Fig 4a), SC205 (Fig 4b) and SC8 (Fig 4c) respectively. A total of 122 spots common to W14, SC205 and SC8 were detected (Fig 5a). They were classified according to gene ontology (Fig 5b), and listed in Table 3 and S2 Fig. As a 1.2–1.5-fold change threshold has been often used [7, 18], a 1.5-fold change in pairwise comparison of SC205/W14 and SC8/W14 was used as significance to assess protein profiles. While 36 and 31 proteins were observed to vary differentially within the pairs for SC205/W14 and SC8/W14, respectively, with greater than ±1.5-fold in all triplicate gels. These included 25 up- and 11 down-regulated in SC205, and 21 up- and 10 down-regulated in SC8 compared to W14 (Table 3). Five common proteins were detected in both SC8 and W14 leaves, 10 common proteins between SC205 and W14, and 23 common proteins in both SC205 and SC8 leaves (Fig 5a). Additionally, 11, 2 and 2 spots were unique to W14, SC205 and SC8, respectively (Fig 5a, S3 Fig and Table 4).

**Table 3:** Identification of 122 common proteins in leaves of SC205, SC8 and W14. The spots showing the similar proteins from 2-DE images of cassava SC205, SC8 and W14 leaves, and the number were counted after gel analysis and manual editing with Delta 2D software.

Spot Number^a^	Identification	Accession no^b^	Theoretical pI/Mw(kDa)	Fold changes SC205/W14^c^	Fold changes SC8/W14^d^
*Photosynthesis related proteins (24)*					
1**	nuclear encoded precursor to chloroplast protein—*Pisum sativum*	AAA33680	6.55/102.71	3.45±0.23(+)	3.52±0.28(+)
18**	rubisco subunit binding-protein alpha subunit, ruba, putative—*Ricinus communis*	EEF28034	5.25/53.20	2.03±0.11(+)	2.21±0.12(+)
19	rubisco subunit binding-protein alpha subunit, ruba, putative—*R*. *communis*	EEF28034	5.25/53.20	1.45±0.13(+)	1.32±0.10(+)
31	ribulose-bisphosphate carboxylase, large subunit—*Krameria lanceolata*	CAA75263	7.29/40.39	1.45±0.13(+)	1.32±0.10(+)
32**	ribulose-1,5-bisphosphate carboxylase/oxygenase large subunit—*Kunhardtia radiata*	AAD02082	6.13/52.10	3.68±0.24(+)	3.88±0.26(+)
36**	putative RuBisCo activase protein—*Zantedeschia hybrid cultivar*	AAT12492	5.08/27.69	2.92±0.20(+)	3.36±0.22(+)
37**	putative RuBisCo activase protein—*Z*. *hybrid cultivar*	AAT12492	5.08/27.69	2.02±0.20(+)	2.08±0.17(+)
38**	ribulose-1,5-bisphosphate carboxylase/oxygenase activase 2—*Gossypium hirsutum*	AAG61121	5.06/48.35	2.89±0.19(+)	2.91±0.23(+)
40**	ribulose bisphosphate carboxylase activase—*Nicotiana tabacum*	CAA78702	4.83/22.98	3.52±0.26(+)	3.64±0.30(+)
42	ribulose bisphosphate carboxylase activase—*N*. *tabacum*	CAA78702	4.83/22.98	1.21±0.10(+)	1.10±0.09(+)
52	ribulose bisphosphate carboxylase activase—*N*. *tabacum*	CAA78702	4.83/22.98	1.10±0.09(+)	1.12±0.11(+)
53	ribulose-1,5-bisphosphate carboxylase/oxygenase activase 2—*G*. *hirsutum*	AAG61121	5.06/48.35	1.09±0.06(+)	1.11±0.12(+)
60*	ribulose-1,5-bisphosphate carboxylase/oxygenase large subunit, partial (chloroplast)—*Fissistigma polyanthoides*	AFM94281	6.13/49.71	1.86±0.13(-)	1.08±0.10(+)
62**	ribulose-bisphosphate carboxylase, large subunit—*K*. *lanceolata*	CAA75263	7.29/40.39	3.94±0.24(+)	4.65±0.28(+)
68**	Phosphoribulokinase precursor—*A*. *thaliana*	AAG50797	5.71/44.46	4.66±0.27(+)	4.01±0.20(+)
71	Phosphoribulokinase, chloroplastic	P27774	6.03/44.11	1.06±0.08(+)	1.12±0.09(+)
85	Photosystem II stability/assembly factor HCF136, chloroplast precursor, putative—*R*. *communis*	XP_002520925	7.11/43.44	1.35±0.16(-)	1.01±0.10(+)
105**	ribulose-5-phosphate-3-epimerase, putative—*R*. *communis*	EEF47836	9.71/28.21	2.02±0.11(+)	2.06±0.13(+)
112	chloroplast ribose-5-phosphate isomerase—*Spinacia oleracea*	AAL77589	6.54/30.86	1.36±0.09(+)	1.40±0.11(+)
113**	chloroplast ribose-5-phosphate isomerase—*S*. *oleracea*	AAL77589	6.54/30.86	2.06±0.12(-)	2.10±0.15(-)
126	Oxygen-evolving enhancer protein 2, chloroplastic	P16059	8.28/28.05	1.02±0.08(-)	1.02±0.07(+)
127*	Oxygen-evolving enhancer protein 2, chloroplast precursor, putative—*R*. *communis*	XP_002521576	8.63/28.60	1.92±0.13(+)	1.10±0.09(+)
134	Cytochrome b6-f complex iron-sulfur subunit, chloroplastic	P26291	8.63/24.24	1.06±0.10(+)	1.11±0.12(+)
141	ribulose bisphosphate carboxylase activase—*N*. *tabacum*	CAA78702	4.83/22.98	1.10±0.09(+)	1.00±0.10(+)
*Carbohydrate and energy metabolism associated proteins (31)*					
2**	transketolase, putative—*R*. *communis*	EEF50359	6.99/81.52	5.36±0.31(+)	5.42±0.29(+)
14**	transketolase, putative -*R*. *communis*	EEF50359	6.99/81.52	1.65±0.16(+)	1.52±0.18(+)
15	ATP synthase subunit beta, mitochondrial	P29685	6.31/60.33	1.06±0.08(+)	1.08±0.10(+)
22	ATP synthase subunit beta, chloroplastic	P26530	5.15/53.47	1.01±0.11(+)	1.00±0.09(+)
23	ATP synthase subunit beta, chloroplastic	P26530	5.15/53.47	1.00±0.09(+)	1.12±0.11(+)
24**	ATP synthase subunit beta, chloroplastic	P26530	5.15/53.47	1.68±0.17(+)	1.77±0.20(+)
25**	ATP synthase subunit beta, chloroplastic	P26530	5.15/53.47	1.80±0.18(+)	1.92±0.22(+)
26**	ATP synthase subunit beta, mitochondrial	P17614	5.95/59.86	2.08±0.14(-)	2.12±0.18(-)
27	ATP synthase subunit alpha, chloroplastic	B1NWD5	4.93/55.62	1.22±0.09(+)	1.19±0.11(+)
28**	ATP synthase subunit beta, mitochondrial	P17614	5.95/59.86	2.03±0.19(-)	1.98±0.16(-)
29**	ATP synthase subunit beta, mitochondrial	P17614	5.95/59.86	2.06±0.18(+)	2.12±0.20(+)
30	ATP synthase subunit beta, mitochondrial	P17614	5.95/59.86	1.09±0.09(+)	1.15±0.10(+)
49**	alcohol dehydrogenase, putative—*R*. *communis*	XP_002525379	8.61/41.58	2.13±0.20(-)	2.20±0.18(-)
50**	ATP synthase subunit beta, mitochondrial	P29685	6.31/60.34	1.88±0.13(-)	1.90±0.15(-)
51	Enolase 2	Q9LEI9	5.92/47.91	1.14±0.08(+)	1.08±0.10(+)
59	phosphoglycerate kinase—*A*. *thaliana*	AAB60303	4.93/41.91	1.33±0.16(-)	1.27±0.11(-)
61	phosphoglycerate kinase, putative—*R*. *communis*	EEF48756	9.22/50.11	1.04±0.08(+)	1.06±0.10(+)
65**	malate dehydrogenase, putative *-R*. *communis*	EEF38101	8.57/36.31	2.12±0.13(+)	2.20±0.19(+)
67	FtsZ protein—*P*. *sativum*	CAA75603	7.73/44.44	1.12±0.09(+)	1.23±0.12(+)
74**	alcohol dehydrogenase, putative—*R*. *communis*	XP_002525379	8.61/41.58	1.62±0.18(-)	1.50±0.14(-)
82	ATP synthase beta subunit—*Gunnera manicate*	ABV65134	5.23/54.10	1.08±0.12(+)	1.19±0.15(+)
83	beta-carotene hydroxylase 1—*Zea mays*	ACX49217	11.85/11.49	1.12±0.16(-)	1.08±0.10(-)
99	NAD(P)-binding Rossmann-fold-containing protein—*A*. *thaliana*	NP_565868	8.37/34.88	1.10±0.12(+)	1.12±0.09(-)
103	Carbonic anhydrase—*Flaveria brownii*	AAA86942	5.70/35.55	1.33±0.15(+)	1.21±0.11(+)
108**	Carbonic anhydrase, chloroplastic	P16016	6.61/34.57	2.02±0.19(-)	1.88±0.16(-)
109**	Carbonic anhydrase, chloroplastic	P16016	6.61/34.57	1.89±0.17(-)	1.78±0.15(-)
114	putative triosephosphate isomerase—*A*. *thaliana*	AAD29799	7.67/33.35	1.03±0.11(+)	1.05±0.14(+)
115	Putative ATP-binding protein—*Stenotrophomonas maltophilia K279a*	CAQ46869	5.87/30.40	1.06±0.12(+)	1.17±0.14(+)
130	ATP synthase D chain, mitochondrial, putative—*R*. *communis*	XP_002526342	5.21/19.84	1.18±0.16(-)	1.12±0.15(-)
142	ATP synthase epsilon chain—*Androya decaryi*	CAD22407	5.87/14.28	1.09±0.12(-)	1.01±0.10(+)
143	ATP synthase CF1 epsilon subunit—*M*. *esculenta*	YP_001718443	5.18/14.65	1.23±0.15(+)	1.26±0.15(+)
*Detoxifying and antioxidant (8)*					
96	ascorbate peroxidase APX2—*M*. *esculenta*	AAX84679	5.31/27.67	1.26±0.10(-)	1.39±0.12(-)
97**	ascorbate peroxidase APX2—*M*. *esculenta*	AAX84679	5.31/27.67	2.68±0.17(-)	2.66±0.18(-)
116	ascorbate peroxidase APX2—*M*. *esculenta*	AAX84679	5.31/27.67	1.18±0.11(-)	1.12±0.10(-)
119	superoxide dismutase [fe], putative—*R*. *communis*	XP_002511050	4.84/34.50	1.26±0.15(+)	1.28±0.16(+)
120	peroxiredoxin—*Phaseolus vulgaris*	CAC17803	5.18/28.62	1.20±0.14(+)	1.23±0.18(+)
122	2-cys peroxiredoxin-like protein—*Hyacinthus orientalis*	AAT08751	4.93/21.86	1.03±0.09(+)	1.02±0.11(+)
123	2-cys peroxiredoxin like protein—*H*. *orientalis*	AAT08751	4.93/21.86	1.09±0.10(+)	1.11±0.12(+)
124	Peroxiredoxins, putative -*R*. *communis*	EEF32207	8.61/29.40	1.17±0.13(-)	1.08±0.08(-)
*Protein biosynthesis (9)*					
57	choloroplast translation elongation factor—*P*. *sativum*	CAA74893	6.62/53.05	1.04±0.12(+)	1.00±0.10(+)
58	Elongation factor Tu, chloroplastic	O24310	6.62/53.05	1.12±0.09(+)	1.20±0.11(+)
86	elongation factor-1 beta A1—*A*. *thaliana*	CAA52751	4.50/25.20	1.18±0.13(-)	1.15±0.10(-)
87	elongation factor-1 beta A1—*A*. *thaliana*	CAA52751	4.50/25.20	1.19±0.11(+)	1.21±0.14(+)
106	50S ribosomal protein L4, putative—*R*. *communis*	XP_002525600	8.64/31.21	1.26±0.17(-)	1.14±0.12(-)
110	Proteasome subunit alpha type-5	Q9M4T8	4.70/25.98	1.05±0.09(+)	1.06±0.10(-)
111	ATP-dependent Clp protease proteolytic subunit (ClpP4)—*A*. *thaliana*	AAM65254	5.37/31.51	1.03±0.12(-)	1.04±0.11(-)
121	30S ribosomal protein S8, chloroplastic	Q2WGF1	11.18/14.52	1.09±0.13(-)	1.10±0.14(-)
138	50S ribosomal protein L12, chloroplastic	P84558	4.25/1.49	1.12±0.11(+)	1.08±0.12(-)
*Chaperones (10)*					
3**	heat shock protein 82 (HSP82)—*Oryza sativa*	CAA77978	4.99/80.19	3.42±0.22(+)	1.95±0.15(+)
4	HSP90-1- *Glycine max*	ADC45395	4.94/80.38	1.25±0.11(+)	1.33±0.14(+)
5	heat shock protein 70—*Cucumis sativus*	CAA52149	5.15/75.41	1.05±0.09(+)	1.08±0.11(+)
8	hsp70 (AA 6–651)—*Petunia× hybrida*	CAA31663	5.06/70.78	1.14±0.10(+)	1.06±0.09(+)
11	Heat shock 70 kDa protein, mitochondrial	Q01899	5.95/72.54	1.10±0.08(+)	1.04±0.10(+)
54	SHOOT1 protein—*G*. *max*	AAK37555	5.26/40.24	1.02±0.09(+)	1.04±0.11(+)
81	Embryonic abundant protein VF30.1 precursor, putative—*R*. *communis*	EEF39169	6.25/24.95	1.16±0.11(+)	1.18±0.15(+)
84	SHOOT1 protein—*G*. *max*	AAK37555	5.26/40.24	1.03±0.08(+)	1.05±0.10(+)
107	binding / catalytic/ coenzyme binding—*A*. *thaliana*	NP_565868	8.37/34.88	1.02±0.07(+)	1.08±0.12(+)
139	HSP19 class II—*Citrus× paradisi*	AAP33012	8.01/11.14	1.21±0.13(+)	1.13±0.11(-)
*HCN metabolism (4)*					
6*	linamarase—*M*. *esculenta*	AAB22162	5.52/61.37	2.21±0.18(+)	1.05±0.12(-)
7	linamarase—*M*. *esculenta*	AAB22162	5.52/61.37	1.12±0.09(+)	1.06±0.14(+)
9	linamarase—*M*. *esculenta*	AAB22162	5.52/61.37	1.06±0.10(+)	1.11±0.12(+)
10	linamarase—*M*. *esculenta*	AAB22162	5.52/61.37	1.18±0.09(+)	1.21±0.18(+)
*Structure (4)*					
34	beta-tubulin—*Lotus corniculatus*	AAV71172	5.03/46.89	1.14±0.11(+)	1.02±0.09(-)
35	beta-tubulin—*Lotus corniculatus*	AAV71172	5.03/46.89	1.15±0.12(+)	1.09±0.10(-)
55	Actin-1	P23343	5.64/41.99	1.12±0.09(-)	1.10±0.11(-)
56	actin—*Isatis tinctoria*	AAW63030	5.31/41.82	1.10±0.09(+)	1.13±0.10(+)
*Signal transduction mechanisms (3)*					
20**	Ethylene receptor 1- *Brassica oleracea*	O49230	7.98/82.24	1.88±0.13(+)	1.90±0.14(+)
21	Calcium and intergrin bingding 1(calmyin)), isoform CRA_b—*Homo sapiens*	EAX02088	4.71/23.07	1.44±0.14(+)	1.33±0.11(+)
89	14-3-3 protein—*M*. *esculenta*	ADD92154	4.79/29.81	1.12±0.09(+)	1.06±0.10(+)
*Amino acid metabolism (4)*					
41	amino acid binding protein, putative—*R*. *communis*	EEF39366	5.59/30.92	1.08±0.10(+)	1.06±0.09(+)
44**	S-adenosylmethionine synthase 2—*A*. *thaliana*	NP_192094	5.67/43.26	1.82±0.16(+)	1.74±0.15(+)
75	cysteine synthase—*N*. *tabacum*	CAC12819	5.84/34.96	1.38±0.12(+)	1.46±0.16(+)
140	mitochondrial glycine decarboxylase complex H-protein—*Populus tremuloides*	ABO61731	4.78/17.62	1.12±0.10(-)	1.09±0.09(-)
*Defense (8)*					
13*	Beta-glucosidase—*M*. *esculenta*	CAA64442	5.80/63.10	1.62±0.14(+)	1.26±0.10(+)
72	poly(A) polymerase—*P*. *sativum*	AAC50041	5.33/50.23	1.12±0.09(-)	1.08±0.06(-)
77**	plastidic aldolase—*Nicotiana paniculata*	BAA77603	6.38/42.82	2.13±0.16(+)	1.86±0.15(+)
80**	plastidic aldolase—*Nicotiana paniculata*	BAA77603	6.38/42.82	3.86±0.24(+)	3.19±0.22(+)
100	Chloroplast Drought-induced Stress Protein of 32kDa—*Solanum tuberosum*	CAA71103	8.07/33.46	1.16±0.10(-)	1.08±0.11(-)
101	chloroplast latex aldolase-like protein—*M*. *esculenta*	AAV74407	6.22/33.81	1.42±0.15(-)	1.37±0.12(-)
117	chloroplast latex aldolase-like protein—*M*. *esculenta*	AAV74407	6.54/34.02	1.22±0.10(-)	1.18±0.09(-)
118	chloroplast latex aldolase-like protein—*M*. *esculenta*	AAV74407	6.54/34.02	1.13±0.09(-)	1.06±0.08(-)
*Inorganic ion transport and metabolism (1)*					
33	calreticulin—*A*. *thaliana*	AAA80652	4.37/46.58	1.06±0.11(-)	1.28±0.13(-)
*DNA binding proteins (1)*					
128	DNA-binding protein—*Z*. *mays*	CAA46876	5.87/18.29	1.06±0.11(+)	1.02±0.11(+)
*Function unknown proteins (15)*					
12	unnamed protein product—*M*. *esculenta*	CBV34462	5.53/61.45	1.56±0.13(+)	1.15±0.12(+)
43	predicted protein—*Physcomitrella patens subsp*. *patens*	EDQ51995	6.02/52.39	1.38±0.12(+)	1.45±0.14(+)
69	predicted protein—*Physcomitrella patens subsp*. *patens*	EDQ53885	6.76/46.38	1.06±0.09(+)	1.11±0.12(+)
76	Unknown—*Medicago truncatula*	ACJ84643	8.33/41.19	1.03±0.11(+)	1.05±0.10(+)
88	Predicted protein—*Hordeum vulgare subsp*. *vulgare*	BAK02253	4.62/29.03	1.07±0.13(+)	1.05±0.09(+)
92	unnamed protein product—*S*. *oleracea*	CAA29062	5.58/35.04	1.42±0.15(-)	1.40±0.13(-)
93	unnamed protein product—*S*. *oleracea*	CAA29062	5.58/35.04	1.06±0.13(-)	1.10±0.12(+)
94	unnamed protein product—*S*. *oleracea*	CAA29062	5.58/35.04	1.08±0.10(-)	1.01±0.09 (-)
95	unnamed protein product—*M*. *esculenta*	CBC70131	5.31/27.67	1.06±0.11(-)	1.12±0.13(+)
102	Pentatricopeptide repeat superfamily protein isoform 1—*Theobroma cacao*	EOY00680	7.76/57.86	1.18±0.11(-)	1.20±0.10(-)
104	unnamed protein product—*M*. *esculenta*	CBV34462	5.53/61.45	1.06±0.10(-)	1.02±0.09(-)
131	predicted protein—*P*. *trichocarpa*	XP_002325568	9.02/26.95	1.10±0.09(+)	1.05±0.08(-)
133**	conserved hypothetical protein—*R*. *communis*	EEF33941	4.81/16.92	1.97±0.13(-)	1.54±0.09(-)
137	conserved hypothetical protein—*R*. *communis*	EEF25206	9.91/30.77	1.12±0.10(+)	1.09±0.11(+)
147	Os07g0469100—*O*.*sativa Japonica Group*	NP_001059599	9.37/15.73	1.07±0.12(+)	1.11±0.09(+)
The total protein number			122		

^a^, The numbers corresponded to the 2-DE gels in Fig 4;

^b^, NCBI accession number;

^c^, Fold changes of protein spots between SC205 and W14 (Values were means ± SE);

^d^, Fold changes of protein spots between SC8 and W14 (Values were means ± SE);

(+) means up-regulated compare with W14, while (-) means down-regulated compare with W14;

* indicates differential protein spots in pairwise comparison of SC205/W14 or SC8/W14;

** indicates differential protein spots in pairwise comparison of SC205/W14 and SC8/W14.

**Table 4:** Identification of the unique protein spots in leaves detected by pairwise comparison of W14/SC205, W14/SC8 and SC205/SC8.

Spot Number^a^	Identification	Accession no^b^	Theoretical pI/Mw(kDa)	Score^c^/ No. of Unique peptides matched^d^
*W14 (11)*				
*Photosynthesis related proteins (1)*				
46	ribulose 1,5-bisphosphate carboxylase/oxygenase large subunit—*Noteroclada confluens*	ADP76575	5.99/47.95	97/1
*Carbohydrate and energy metabolism associated proteins (1)*				
17	ATP synthase subunit beta, mitochondrial	P29685	6.31/60.33	195/1
*Detoxifying and antioxidant (1)*				
90	lactoylglutathione lyase, putative—*R*. *communis*	XP_002518470	7.63/31.55	121/1
*Amino acid metabolism (1)*				
45	Glutamate-1-semialdehyde 2, 1-aminomutase, chloroplastic	P31593	6.43/50.88	78/1
*Structure (1)*				
132	kinesin heavy chain, putative—*R*. *communis*	EEF30221	8.58/99.95	41/1
*Function unknown proteins (6)*				
16	conserved hypothetical protein—R. communis	EEF44175	7.47/18.96	57/1
91	hypothetical protein VITISV_027630—*Vitis vinifera*	CAN61828	5.87/33.23	110/1
135	unknown—*Populus trichocarpa*	ABK95882	6.67/16.59	70/1
136	conserved hypothetical protein -*R*. *communis*	EEF45827	4.51/8.98	38/1
145	conserved hypothetical protein—*R*. *communis*	EEF22198	6.24/17.27	50/1
146	conserved hypothetical protein—*R*. *communis*	EEF22198	6.24/17.27	51/1
*SC205 (2)*				
*Carbohydrate and energy metabolism associated proteins (4)*				
150	phosphoglycerate kinase—*A*. *thaliana*	AAB60303	4.93/41.91	300/3
*Protein biosynthesis (1)*				
163	50S ribosomal protein L12, chloroplastic	P84558	4.25/1.49	86/1
*SC8 (2)*				
*Photosynthesis related proteins (2)*				
174	ribulose-bisphosphate carboxylase, large subunit—*K*. *lanceolata*	CAA75263	7.29/40.39	161/2
175	ribulosebisphosphate carboxylase—*Pandanus tectorius*	AAA68039	6.34/51.69	102/1
*SC205 and SC8 (23)*				
*Photosynthesis related proteins (9)*				
152	ribulose 1,5-bisphosphate carboxylase small chain precursor—*M*. *esculenta*	AAF06098	8.33/20.41	68/1
158	Oxygen-evolving enhancer protein 2, chloroplastic	P16059	8.28/28.05	47/2
161	Oxygen-evolving enhancer protein 2, chloroplastic	P16059	8.28/28.05	130/1
166	ribulose 1,5-bisphosphate carboxylase small chain precursor—*M*. *esculenta*	AAF06098	8.33/20.41	120/3
168	ribulose 1,5-bisphosphate carboxylase small chain precursor—*M*. *esculenta*	AAF06098	8.33/20.41	67/1
169	ribulose 1,5-bisphosphate carboxylase small chain precursor—*M*. *esculenta*	AAF06098	8.33/20.41	175/3
171	ribulose 1,5-bisphosphate carboxylase small chain precursor—*M*. *esculenta*	AAF06098	8.33/20.41	97/2
172	ribulose 1,5-bisphosphate carboxylase small chain precursor—*M*. *esculenta*	AAF06098	8.33/20.41	218/3
173	photosystem I subunit VII—*Pinus thunbergii*	NP_042492	6.68/9.01	47/1
*Carbohydrate and energy metabolism associated proteins (4)*				
151	alcohol dehydrogenase, putative—*R*. *communis*	EEF37017	8.61/41.58	201/3
154	enoyl-ACP reductase—*Oryza sativa (japonica cultivar-group)*	CAA05816	9.10/39.90	148/1
156	Carbonic anhydrase, chloroplastic	P16016	6.61/34.57	147/1
167	ATP synthase CF1 epsilon subunit—*S*. *oleracea*	NP_054942	6.59/14.70	124/1
*Detoxifying and antioxidant (4)*				
160	peroxiredoxin—*Ipomoea batatas*	AAP42502	8.80/20.77	129/1
162	Chain A, Prx D (Type Ii)—*Populus Tremula*	1TP9_A	5.56/17.43	130/2
164	Superoxide dismutase [Cu-Zn], chloroplastic	O65175	6.17/22.08	65/1
170	Glutaredoxin	O81187	6.05/11.13	63/1
*Chaperones (1)*				
149	chaperonin precursor—*Pisum sativum*	AAA66365	5.85/62.98	77/1
*Structure (1)*				
165	Similar to actin binding protein; F6N23.12—*A*. *thaliana*	AAC13618	5.12/15.70	44/1
*HCN metabolism (2)*				
153	acetone-cyanhydrin lyase (EC 4.1.2.37)—cassava	S45682	6.15/29.50	179/3
155	acetone-cyanhydrin lyase (EC 4.1.2.37)—cassava	S45682	6.15/29.50	197/3
*Function unknown proteins (2)*				
157	Predicted protein-Populus trichocarpa	XP_002325568	9.02/26.95	56/1
159	forkhead-associated domain-containing protein—*Arabidopsis lyrata subsp*. *lyrata*	XP_002878556	8.46/22.23	88/1
*SC205 and W14 (10)*				
*Photosynthesis related proteins (2)*				
47	Ferredoxin-NADP reductase, chloroplastic	P41343	8.54/41.06	63/1
63	putative RuBisCo activase protein—*Z*. *hybrid cultivar*	AAT12492	5.08/27.69	80/1
*Carbohydrate and energy metabolism associated proteins (4)*				
48	ATP synthase subunit beta, mitochondrial	P29685	6.31/60.34	131/2
79	ATP binding protein, putative*—R*. *communis*	EEF42393	4.52/95.56	59/1
98	NAD(P)-binding Rossmann-fold-containing protein—*A*. *thaliana*	NP_565868	8.37/34.88	263/2
148	D-cadinene synthase, putative—*R*. *communis*	EEF31472	7.71/14.48	32/1
*Structure(1)*				
39	Alpha-tubulin 3—*Z*. *mays*	CAA44861	5.09/49.56	101/3
*Protein biosynthesis (3)*				
78	conserved hypothetical protein—*R*. *communis*	EEF22968	10.30/33.40	74/1
129	forkhead-associated domain-containing protein—*Arabidopsis lyrata subsp*. *lyrata*	XP_002878556	8.46/22.23	88/1
144	conserved hypothetical protein—*R*. *communis*	EEF24864	4.3/14.02	54/1
*SC8 and W14 (5)*				
*Photosynthesis related proteins (1)*				
70	Phosphoribulokinase, chloroplastic	P27774	6.03/44.11	80/1
*Carbohydrate and energy metabolism associated proteins (1)*				
64	malate dehydrogenase, putative—*R*. *communis*	EEF38101	8.57/36.31	178/1
*Amino acid metabolism (1)*				
73	glutamate-ammonia ligase (EC 6.3.1.2), cytosolic—*A*. *thaliana*	S18603	5.40/40.73	237/2
*Protein biosynthesis (1)*				
66	Late embryogenesis abundant protein Lea14-A, putative—*R*. *communis*	XP_002533345	4.64/34.71	142/2
*Function unknown proteins (1)*				
125	unknown—*P*. *trichocarpa*	ABK94443	6.61/30.03	82/1
The total protein number			53	

^a^, The numbers corresponded to the 2-DE gels in Fig 4 and S3 Fig;

^b^, NCBI accession number;

^c^, Probability-based MOWSE (molecular weight search) scores;

^d^, The number of unique peptides identified by MS/MS, and individual ions scores are all identity or extensive homology (p<0.05).

### Storage root protein profiles

**Fig 6:**
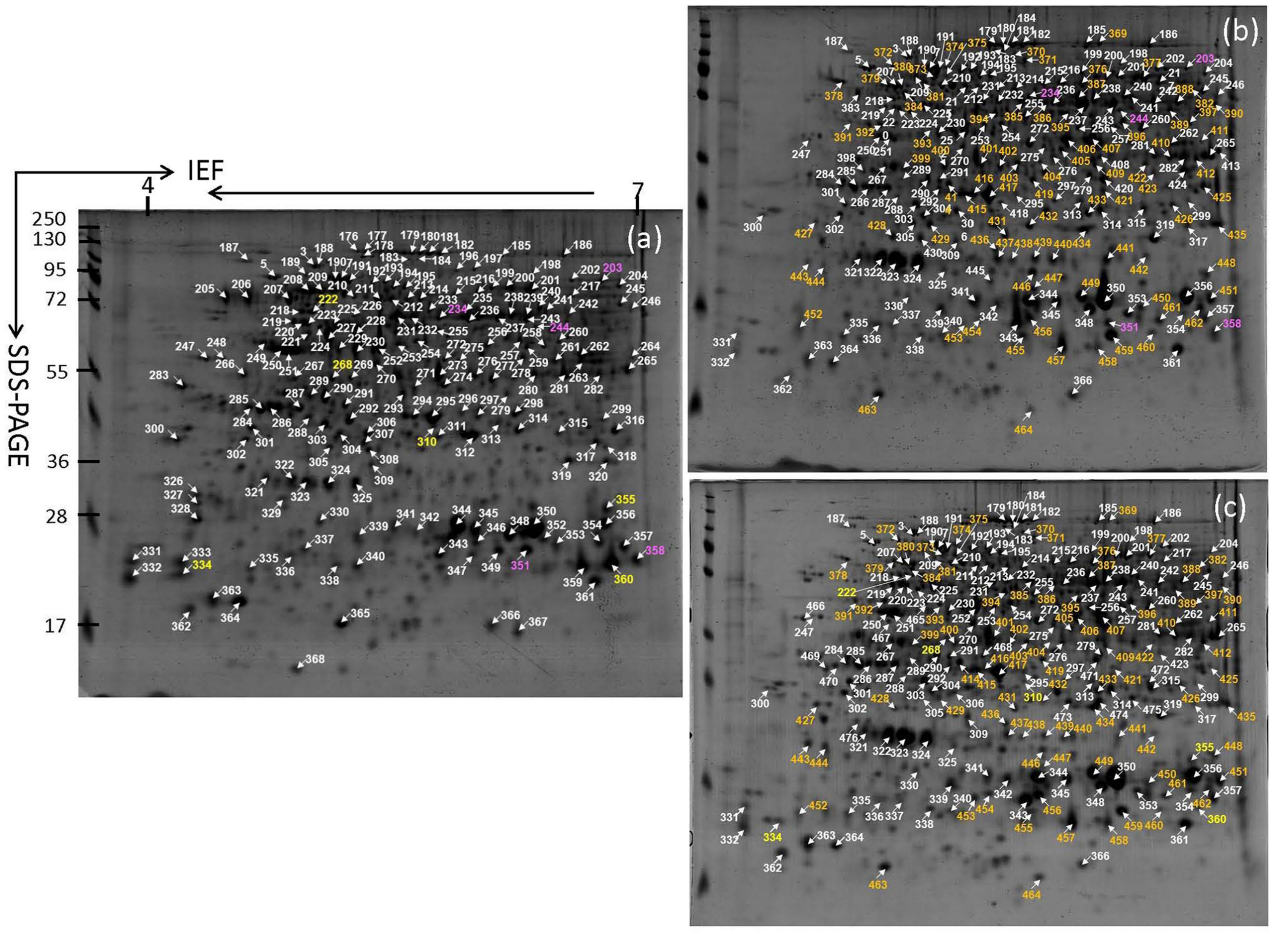
196, 228 and 232 proteins identified by MALDI-TOF-TOF-MS/MS in 2-D gel protein profiles of W14(a), SC205(b) and SC8(c) storage roots, respectively. The pink numbers are common proteins to W14 and SC205, the yellow numbers are common proteins to W14 and SC8, and the orange numbers are common proteins to SC205 and SC8.

**Fig 7:**
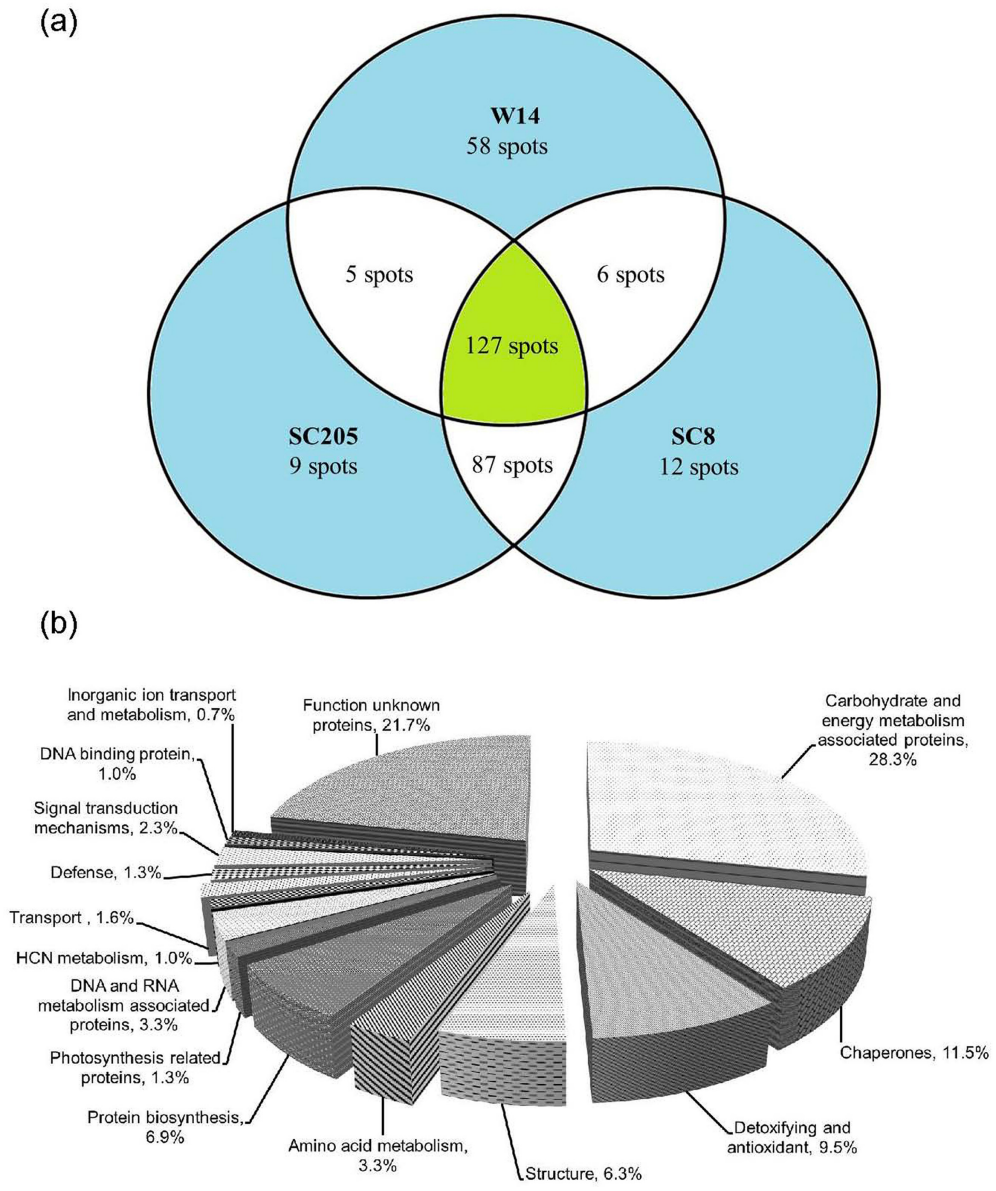
Venn diagrams of 308 proteins identified (a) and their functional classification (b) in storage roots of SC205, SC8 and W14. Functional categorization was performed according to the MIPS database (http://mips.gsf.de).

At least 300 spots gave reproducible staining patterns for all storage root samples (Fig 6). A total of 196, 228 and 232 protein spots were identified in W14, SC205 and SC8 (Fig 7a), respectively. The identified proteins were annotated according to gene ontology and listed in Fig 7b and Table 5. One hundred and twenty-seven protein spots were common to all samples (Fig 7a and S4 Fig), in which 39 stained spots in SC205 and 40 stained spots in SC8 were found to have significant changes (p<0.05) with greater than 1.5-fold altered intensity compared with W14 in all three biological replicates. Of these, 19 up- and 20 down-regulated proteins in the pairwise comparison of SC205/W14, 18 up- and 22 down-regulated in the comparison of SC8/W14 were shown in Table 6. Five common proteins were detected in storage roots between W14 and SC205, 6 common proteins between W14 and SC8, and 87 common proteins between SC205 and SC8 (Fig 7a, Table 6). In addition, 58, 9 and 12 protein spots were unique to W14, SC205 and SC8, respectively (Fig 7a, S5 Fig and Table 6).

**Table 5:** Identification of 127 common proteins from storage roots of SC205, SC8 and W14. The spots showing the same proteins in storage roots of W14, SC205 and SC8, and the number were counted after gel analysis and manual editing with Delta 2D software.

Spot Number^a^	Identification	Accession no^b^	Theoretical pI/Mw(kDa)	Fold changes SC205/W14^c^	Fold changes SC8/W14^d^
*Carbohydrate and energy metabolism associated proteins (42)*					
179	Starch phosphorylase L; Flags: Precursor	P27598	5.26/108.52	1.05±0.13(+)	1.22±0.18(+)
180	Starch phosphorylase L; Flags: Precursor	P27598	5.26/108.52	1.20±0.11(+)	1.18±0.12(+)
181	Starch phosphorylase L; Flags: Precursor	P27598	5.26/108.52	1.19±0.13(+)	1.09±0.10(+)
182	Starch phosphorylase L; Flags: Precursor	P27598	5.26/108.52	1.06±0.10(+)	1.14±0.10(-)
183	NADH-ubiquinone oxidoreductase, putative—*R*. *communis*	XP_002531931	6.56/80.77	1.38±0.20(+)	1.40±0.22(+)
184	NADH-ubiquinone oxidoreductase, putative—*R*. *communis*	XP_002531931	6.56/80.77	1.40±0.19(+)	1.41±0.18(+)
185**	aconitase—*A*. *thaliana*	CAA58046	5.98/100.82	1.89±0.21(+)	1.58±0.15(+)
187	Aldo-keto reductase, putative—*R*. *communis*	XP_002521902	6.22/36.64	1.19±0.13(+)	1.34±0.16(+)
194	V-type proton ATPase catalytic subunit A	P09469	5.29/68.84	1.06±0.10(+)	1.04±0.10(+)
195	ATP synthase alpha subunit vacuolar, putative—*R*. *communis*	EEF48722	6.04/25.02	1.03±0.09(+)	1.08±0.13(+)
198	NADH-ubiquinone oxidoreductase, putative—*R*. *communis*	XP_002531931	6.56/80.77	1.02±0.08(+)	1.10±0.11(+)
199	phosphoglucomutase, putative—*R*. *communis*	XP_002527783	5.53/63.25	1.13±0.11(+)	1.18±0.15(+)
200**	Succinate dehydrogenase flavoprotein subunit—*P*. *trichocarpa*	XP_002310225	6.40/69.86	1.59±0.13(+)	1.64±0.16(+)
212	ADP glucose pyrophosphorylase small subunit 1-like protein—*Malus × domestica*	ADG27450	7.11/56.52	1.36±0.15(-)	1.24±0.12(-)
213	ATP synthase subunit beta, mitochondrial-like—*Brachypodium distachyon*	XP_003567942	59.12/6.00	1.10±0.08(-)	1.08±0.09(-)
215	d-3-phosphoglycerate dehydrogenase, putative—*R*. *communis*	XP_002518687	7.65/63.10	1.23±0.15(+)	1.16±0.10(+)
216	d-3-phosphoglycerate dehydrogenase, putative—*R*. *communis*	XP_002518687	7.65/63.10	1.10±0.09(+)	1.02±0.07(+)
217*	ATP synthase subunit beta, mitochondrial;	P17614	5.95/59.86	1.55±0.16(+)	1.34±0.16(-)
218	ATP synthase subunit beta vacuolar, putative—*R*. *communis*	XP_002510596	4.99/54.35	1.08±0.08(-)	1.10±0.11(-)
224	Enolase 2	Q9LEI9	5.92/47.91	1.03±0.09(+)	1.06±0.12(+)
231**	ATP synthase beta subunit, putative—*R*. *communis*	XP_002532227	6.00/59.93	1.66±0.17(-)	1.54±0.12(-)
236	Enolase 2	Q9LEI9	5.92/47.91	1.46±0.15(-)	1.38±0.10(-)
238	Enolase 2	Q9LEI9	5.92/47.91	1.08±0.09(+)	1.12±0.08(+)
241**	Enolase 2	Q9LEI9	5.92/47.91	2.15±0.20(+)	1.74±0.16(+)
243**	Enolase 2	Q9LEI9	5.92/47.91	2.38±0.18(+)	2.40±0.21(+)
245	dihydrolipoamide dehydrogenase precursor—*Bruguiera gymnorhiza*	BAB44156	6.71/53.97	1.36±0.11(+)	1.28±0.10(+)
251	pyruvate dehydrogenase, putative—*R*. *communis*	XP_002512633	5.95/39.45	1.06±0.08(+)	1.08±0.10(+)
255**	ADPglucose pyrophosphorylase—*O*. *sativa*	AAA33891	5.65/53.51	3.58±0.18(+)	2.46±0.12(+)
256	ATP synthase subunit beta, mitochondrial; Flags: Precursor	P17614	5.95/59.86	1.08±0.09(-)	1.10±0.10(-)
257	phosphoglycerate kinase, putative—*R*. *communis*	XP_002513352	5.65/42.39	1.12±0.10(-)	1.16±0.14(-)
260**	dihydrolipoyllysine-residue succinyltransferase component of 2-oxoglutarate dehydrogenase complex—*Z*. *mays*	ACG35848	8.22/48.68	2.42±0.20(+)	1.66±0.18(+)
262**	sinapyl alcohol dehydrogenase—*P*. *tremuloides*	AAK58693	6.23/38.99	2.86±0.21(-)	4.12±0.22(-)
270	UDP-glucosyltransferase, putative—*R*. *communis*	XP_002518353	5.35/39.75	1.32±0.12(-)	1.06±0.10(-)
275	sinapyl alcohol dehydrogenase—*P*. *tremuloides*	AAK58693	6.23/38.99	1.07±0.09(-)	1.09±0.10(-)
276	fructose-bisphosphate aldolase, putative—*R*. *communis*	XP_002526308	8.64/42.79	1.12±0.10(-)	1.05±0.08(-)
279**	Protein yrdA, putative—*R*. *communis*	XP_002510412	5.78/29.50	1.57±0.13(+)	1.63±0.18(+)
287	putative inorganic pyrophosphatase—*Oryza sativa Japonica Group*	BAD16934	5.80/31.78	1.02±0.07(+)	1.30±0.11(+)
304	putative triosephosphate isomerase—*A*. *thaliana*	AAD29799	7.67/33.35	1.25±0.14(+)	1.16±0.12(+)
314	triosephosphate isomerase—*Glycine max*	AAT46998	5.87/27.23	1.18±0.09(-)	1.02±0.08(-)
321	pheophorbide A oxygenase, putative—*R*. *communis*	XP_002523735	6.58/60.28	1.10±0.08(+)	1.05±0.06(+)
330	ATP synthase D chain, mitochondrial, putative—*R*. *communis*	XP_002529702	5.33/19.74	1.16±0.11(-)	1.08±0.10(-)
344	phosphoenolpyruvate mutase- *Selenomonas sp*. *oral taxon 149 str*. *67H29BP*	ZP_07397771	5.29/48.01	1.36±0.12(-)	1.18±0.10(-)
*Chaperones (20)*					
3	heat shock protein 82 (HSP82)—*O*. *sativa*	CAA77978	4.99/80.19	1.06±0.09(-)	1.18±0.13(+)
5	heat shock protein 70—*Cucumis sativus*	CAA52149	5.15/75.41	1.24±0.12(+)	1.13±0.10(+)
7	hsp70 (AA 6–651)—*Petunia× hybrida*	CAA31663	5.06/70.78	1.07±0.09(+)	1.21±0.14(+)
188	molecular chaperone Hsp90-1—*N*. *benthamiana*	AAR12193	4.93/80.10	1.12±0.10(+)	1.45±0.14(+)
190	Heat Shock 70kD protein—*G*. *max*	CAA44620	5.36/70.88	1.10±0.09(-)	1.08±0.06(-)
192	heat shock protein—*Z*. *mays*	AFW68374	5.62/72.69	1.12±0.07(+)	1.24±0.12(+)
193	heat shock protein, putative—*R*. *communis*	XP_002518324	6.10/71.12	1.06±0.10(-)	1.07±0.08(+)
202	heat shock protein 70 (HSP70)-interacting protein, putative—*R*. *communis*	XP_002509580	5.60/65.14	1.06±0.09(+)	1.02±0.05(+)
210**	chaperonin 60 beta—wheat (fragment)	JT0902	5.10/16.53	3.12±0.16(+)	2.64±0.18(+)
214	chaperonin-60kD, ch60, putative- *R*. *communis*	XP_002518171	5.84/61.34	1.05±0.10(-)	1.38±0.12(-)
281	annexin, putative- *R*. *communis*	XP_002513910	6.81/36.20	1.13±0.11(-)	1.32±0.13(-)
313	groes chaperonin, putative—*R*. *communis*	XP_002516232	8.89/26.60	1.24±0.10(+)	1.06±0.08(-)
317*	Prefoldin subunit, putative—*R*. *communis*	XP_002522938	5.32/14.95	1.36±0.12(-)	1.68±0.15(-)
322**	heat shock protein, putative—*R*. *communis*	XP_002532054	8.59/26.10	5.26±0.23(+)	4.98±0.24(+)
323**	Small heat shock protein isoform 2—*T*. *cacao*	XP_007039186	6.85/25.24	6.12±0.33(+)	5.96±0.28(+)
325**	Small heat shock protein isoform 2—*T*. *cacao*	XP_007039186	6.85/25.24	4.32±0.21(-)	4.30±0.22(-)
336	annexin, putative—*R*. *communis*	XP_002513910	6.81/36.20	1.13±0.11(+)	1.04±0.08(+)
343**	HSP19 class II—*Citrus × paradisi*	AAP33012	5.27/15.82	2.08±0.20(+)	3.79±0.24(+)
350	heat-shock protein, putative- *R*. *communis*	XP_002530396	6.34/18.42	1.15±0.10(+)	1.26±0.14(+)
356**	18.1 kDa class I heat shock protein	P27879	5.20/16.47	1.89±0.21(+)	1.93±0.21(+)
*Detoxifying and antioxidant (9)*					
201**	malic enzyme, putative—*R*. *communis*	XP_002514230	5.98/65.19	3.20±0.23(-)	1.50±0.14(-)
232	Monodehydroascorbate reductase family protein—*P*. *trichocarpa*	XP_006381300	6.51/47.05	1.12±0.09(+)	1.02±0.08(+)
282*	aldo/keto reductase AKR—*M*. *esculenta*	AAX84672	6.38/37.71	2.14±0.15(+)	1.04±0.10(+)
284	dehydrin—*Populus alba*	ABS12345	5.14/25.92	1.13±0.11(-)	1.06±0.08(-)
295**	ascorbate peroxidase APX2—*M*. *esculenta*	AAX84679	5.31/27.67	9.86±0.56(-)	9.72±0.60(-)
299**	ascorbate peroxidase—*C*. *maxima*	ACM17463	5.47/27.51	1.62±0.10(-)	2.05±0.11(-)
303	L-ascorbate peroxidase—*P*. *sativum*	CAA43992	5.52/27.19	1.09±0.08(+)	1.18±0.11(+)
319**	Superoxide dismutase [Mn], mitochondrial	P35017	7.10/25.84	2.26±0.23(+)	1.55±0.14(+)
364**	Thioredoxin h—*H*. *brasiliensis*	AAD33596	4.82/13.85	2.46±0.33(-)	1.62±0.15(-)
*Structure(11)*					
211	Actin-1	P23343	5.64/41.99	1.28±0.12(-)	1.33±0.11(-)
220	Tubulin beta-1 chain	P12411	4.68/50.22	1.30±0.13(-)	1.06±0.10(+)
223	beta-tubulin—*C*. *maxima*	ACM78033	4.82/49.88	1.07±0.09(+)	1.10±0.10(+)
225	alpha-tubulin—*Picea abies*	CAA41045	4.58/12.57	1.40±0.20(-)	1.33±0.14(-)
252	actin family protein—*P*. *trichocarpa*	XP_002298710	5.31/41.70	1.28±0.12(-)	1.19±0.11(-)
253	Actin-1	P23343	5.64/41.99	1.02±0.10(+)	1.06±0.09(+)
265	annexin, putative—*R*. *communis*	EEF48493	6.81/36.20	1.15±0.09(+)	1.08±0.10(+)
292**	DREPP plasma membrane polypeptide family protein—*P*. *trichocarpa*	XP_006385859	4.93/22.10	2.05±0.16(-)	2.58±0.20(-)
305*	Stem-specific protein TSJT1, putative- *R*. *communis*	XP_002517179	5.54/25.34	1.35±0.11(+)	2.08±0.15(-)
362*	Major latex protein, putative—*R*. *communis*	XP_002534267	5.43/16.83	1.56±0.16(-)	1.33±0.14(+)
363	Remorin, putative—*R*. *communis*	XP_002509770	8.60/14.02	1.28±0.11(-)	1.21±0.10(+)
*Amino acid metabolism (2)*					
247**	sensory transduction histidine kinase, putative—*R*. *communis*	XP_002521152	6.52/16.74	2.58±0.19(-)	2.67±0.18(-)
288**	Aspartic proteinase precursor, putative- *R*. *communis*	XP_002529926	5.19/55.83	1.64±0.10(-)	2.10±0.11(-)
*Protein biosynthesis (8)*					
186	elongation factor, partial -*Triticum aestivum*	AAP80650	5.82/18.48	1.18±0.10(+)	1.02±0.06(+)
240	mitochondrial processing peptidase beta subunit—*Cucumis melo*	AAK07827	6.56/58.88	1.19±0.11(+)	1.04±0.07(+)
254	elongation factor Tu, chloroplastic-like—*Vitis vinifera*	XP_002277301	6.24/52.69	1.23±0.12(+)	1.03±0.10(+)
289**	proteasome subunit alpha type-1-A-like—*C*. *sativus*	XP_004152689	4.98/30.88	2.47±0.19(-)	2.40±0.20(-)
302	20S proteasome subunit PAE1—*A*. *thaliana*	AAC32060	4.70/25.95	1.08±0.09(+)	1.10±0.10(+)
306	proteasome subunit beta type-5—*V*. *vinifera*	XP_002264828	6.12/29.27	1.33±0.11(-)	1.26±0.07(-)
342*	initiation factor eIF5-A—*M*. *esculenta*	AAF79401	5.35/21.06	1.16±0.12(+)	2.35±0.15(+)
345	initiation factor eIF5-A—*M*. *esculenta*	AAF79401	5.35/21.06	1.14±0.11(+)	1.20±0.12(+)
*Photosynthesis related proteins (2)*					
242**	Ribulose bisphosphate carboxylase large chain	P28427	6.60/51.81	6.13±0.32(+)	5.67±0.25(+)
291**	Phosphoenolpyruvate carboxylase family protein isoform 5, partial—*T*. *cacao*	XP_007034022	8.85/49.20	2.14±0.17(-)	2.10±0.12(-)
*DNA and RNA metabolism associated proteins (5)*					
237	maturase K- *Armeria gaditana*	AAF76410	9.77/44.46	1.45±0.16(+)	1.39±0.11(+)
335	putative non-LTR retroelement reverse transcriptase—*A*. *thaliana*	AAD22368	9.12/36.19	1.02±0.11(+)	1.04±0.09(+)
353**	glycine-rich RNA-binding protein—*Citrus unshiu*	BAA92156	7.85/16.85	2.76±0.16(+)	1.62±0.10(+)
361	Nucleoside diphosphate kinase B	P47920	6.42/16.20	1.06±0.07(+)	1.01±0.10(+)
366	Nucleoside diphosphate kinase B	P47920	6.42/16.20	1.10±0.09(+)	1.05±0.18(+)
*HCN metabolism (2)*					
191	linamarase—*M*. *esculenta*	AAB22162	5.52/61.37	1.22±0.11(+)	1.36±0.14(+)
209*	linamarase*—M*. *esculenta*	AAB22162	5.52/61.37	1.10±0.09(-)	1.85±0.18(-)
*Transport (2)*					
207	Cytochrome P450 71D10, putative—*T*. *cacao*	XP_007020554	9.21/61.73	1.30±0.11(-)	1.35±0.14(-)
297	cytochrome P450, putative—*R*. *communis*	XP_002526382	9.23/57.64	1.12±0.09(-)	1.30±0.13(-)
*Defense (3)*					
290**	lactoylglutathione lyase, putative—*R*. *communis*	XP_002518470	7.63/31.55	2.30±0.14(-)	1.96±0.14(-)
300**	senescence-associated family protein—*P*. *trichocarpa*	XP_002302279	9.06/16.70	2.08±0.10(-)	2.12±0.13(-)
337	putative disease resistance RPP13-like protein 1-like—*Setaria italica*	XP_004980753	6.41/47.35	1.27±0.10(-)	1.35±0.13(-)
*Signal transduction mechanisms (4)*					
286	14-3-3 protein—*M*. *esculenta*	AAY67798	4.75/29.83	1.42±0.13(-)	1.26±0.10(-)
301	14-3-3 protein, putative—*R*. *communis*	XP_002514016	4.71/28.55	1.10±0.09(-)	1.02±0.06(-)
332**	SAUR family protein—*T*. *cacao*	XP_007011526	9.36/18.41	2.55±0.13(-)	1.96±0.11(-)
340**	Auxin-induced protein X10A, putative- *R*. *communis*	XP_002511675	10.61/19.40	1.56±0.11(-)	1.60±0.10(-)
*Function unknown proteins (17)*					
204*	hypothetical protein POPTR_0005s07010g—*P*. *trichocarpa*	XP_006382884	9.27/14.57	1.02±0.10(-)	2.23±0.14(-)
219*	conserved hypothetical protein—*R*. *communis*	XP_002517995	9.18/9.73	1.88±0.11(-)	1.22±0.06(+)
230	unknown—*P*. *trichocarpa*	ABK93198	8.36/41.05	1.26±0.13(-)	1.30±0.11(-)
246	hypothetical protein—*V*. *vinifera*	XP_002274975	10.42/44.77	1.34±0.11(+)	1.27±0.10(+)
250	unnamed protein product- *V*. *vinifera*	CBI30084	6.98/54.35	1.12±0.10(+)	1.18±0.14(+)
267	Hypothetical protein SORBIDRAFT_08g018560-*Sorghum bicolor*	XP_002442341	9.47/45.53	1.14±0.11(+)	1.22±0.10(+)
285	GF14omega isoform—*A*. *thaliana*	AAA96253	4.71/29.13	1.21±0.13(+)	1.30±0.11(+)
309	predicted protein—*P*. *trichocarpa*	XP_002321135	5.24/26.21	1.04±0.11(+)	1.10±0.12(+)
315**	predicted protein—*P*. *trichocarpa*	XP_002314179	5.35/28.42	1.74±0.12(+)	1.88±0.16(+)
324**	hypothetical protein -*V*. *vinifera*	CAN83772	9.51/25.25	3.11±0.21(+)	3.05±0.24(+)
331**	unnamed protein product -*V*. *vinifera*	CBI26320	7.89/58.95	2.54±0.16(-)	1.85±0.16(-)
338	unnamed protein product, partial -*V*. *vinifera*	CBI32005	9.20/21.71	1.01±0.07(+)	1.14±0.11(+)
339	Os01g0722800—*O*. *sativa Japonica Group*	NP_001044103	5.35/18.06	1.20±0.15(+)	1.23±0.16(+)
341	Os01g0722800—*O*. *sativa Japonica Group*	NP_001044103	5.35/18.06	1.34±0.17(+)	1.29±0.14(+)
348	hypothetical protein ZEAMMB73_092050—*Z*. *mays*	DAA40203	5.16/13.03	1.19±0.12(+)	1.23±0.16(+)
354**	hypothetical protein AMTR_s00096p00110370—*Amborella trichopoda*	XP_006840725	9.13/21.40	2.60±0.20(-)	1.87±0.16(-)
357	conserved hypothetical protein—*R*. *communis*	XP_002534082	5.60/18.28	1.10±0.09(+)	1.09±0.10(+)
The total protein number			127		

^a^. The numbers corresponded to the 2-DE gel in Fig 6;

^b^, NCBI accession number;

^c^, Fold changes of protein spots between SC205 and W14 (Values were means **±** SE);

^d^, Fold changes of protein spots between SC8 and W14 (Values were means **±** SE);

(+) means up-regulated compare with W14, while (-) means down-regulated compare with W14;

* indicates differential protein spots in pairwise comparison of SC205/W14 or SC8/W14;

** indicates differential protein spots in pairwise comparison of SC205/W14 and SC8/W14.

**Table 6:** Identification of the unique proteins in storage roots detected by pairwise comparison of W14/SC205, W14/SC8 and SC205/SC8.

Spot Number^a^	Identification	Accession no^b^	Theoretical pI/Mw(kDa)	Score^c^/ No. of Unique peptides matched^d^
*W14 (58)*				
*Carbohydrate and energy metabolism associated proteins (13)*				
197	NADH-ubiquinone oxidoreductase, putative—*R*. *communis*	XP_002531931	6.56/80.77	30/1
206	Esterase precursor, putative- *R*. *communis*	XP_002517773	4.96/41.02	50/1
226	mitochondrial F1 ATP synthase beta subunit—*A*. *thaliana*	CAC81058	6.53/63.37	463/2
233	ATP synthase subunit beta, mitochondrial	P17614	5.95/59.86	342/2
239	enolase—*Solanum lycopersicum*	CAA41115	5.68/47.80	77/1
258	phosphoglycerate kinase, putative—*R*. *communis*	XP_002513352	5.65/42.39	90/1
273	alcohol dehydrogenase, putative—*R*. *communis*	XP_002529813	6.61/40.96	63/1
274	Isoflavone reductase, putative—*R*. *communis*	XP_002510408	5.25/33.30	67/1
277	Zinc-binding dehydrogenase family protein isoform 1—*T*. *cacao*	XP_007033621	6.02/38.61	55/1
327	pyruvate kinase, putative—*R*. *communis*	XP_002519848	6.26/57.79	62/1
329	Fasciclin-like arabinogalactan protein 8—*A*. *thaliana*	NP_566043	5.43/43.07	46/1
330	ATP synthase D chain, mitochondrial, putative—*R*. *communis*	XP_002529702	5.33/19.74	90/1
347	UDP-glucose 4-epimerase, putative—*R*. *communis*	XP_002529901	8.39/45.83	62/1
*Chaperones (4)*				
196	HSP68	AAB26551	5.20/62.38	142/2
248	AP-4 complex subunit sigma-1, putative- *R*. *communis*	XP_002514188	5.53/16.89	60/1
280	annexin, putative- *R*. *communis*	XP_002513910	6.81/36.20	88/1
283	protein binding protein, putative—*R*. *communis*	XP_002511917	8.59/57.95	66/1
*Detoxifying and antioxidant (4)*				
272	isoflavone reductase homolog Bet v 6.0101 -*Betula pendula*	AAC05116	7.82/33.15	53/1
311	dehydroascorbate reductase, putative—R. communis	XP_002523030	5.78/23.56	66/1
312	dehydroascorbate reductase, putative—R. communis	XP_002523030	5.78/23.56	72/1
359	glutaredoxin, grx, putative—R. communis	XP_002509419	4.89/15.77	80/1
*Protein biosynthesis (6)*				
189	acyl-peptide hydrolase-like—*A*. *thaliana*	BAB09360	5.08/75.42	70/1
208	Ribosomal protein L30 family protein isoform 1—*T*. *cacao*	XP_007020722	11.67/12.42	60/1
221	26S proteasome AAA-ATPase subunit RPT5a	Q9SEI2	4.91/47.48	74/6
266	Late embryogenesis abundant protein, group 2 isoform 1—*T*. *cacao*	XP_007026466	4.69/34.53	80/1
316	Proteasome subunit alpha type-6	O48551	5.83/27.39	281/1
346	60S ribosomal protein L23, putative, expressed—*Oryza sativa Japonica Group*	ABF93885	11.08/11.49	65/1
*Structure (1)*				
308	Stem-specific protein TSJT1, putative—*R*. *communis*	XP_002517179	5.54/25.34	75/1
*Amino acid metabolism (4)*				
229	serine-threonine protein kinase, plant-type, putative—*R*. *communis*	XP_002519152	8.83/39.69	88/1
235	leucine aminopeptidase, putative- *R*. *communis*	XP_002529380	8.09/61.21	66/1
249	Transaldolase, putative- *R*. *communis*	XP_002512678	5.50/47.81	65/1
271	proline iminopeptidase, putative- *R*. *communis*	XP_002522039	6.02/44.56	83/1
*Transport (2)*				
177	heavy metal transporting ATPase—*Chlamydomonas reinhardtii*	XP_001699267	6.80/112.99	49/1
178	transitional endoplasmic reticulum ATPase—*A*. *thaliana*	T48355	5.37/93.62	532/5
*DNA and RNA metabolism associated proteins (2)*				
259	putative non-LTR retroelement reverse transcriptas—*A*. *thaliana*	AAD22368	9.12/36.19	50/1
352	MADS-box transcription factor—*L*. *erinus*	BAI59709	9.40/27.50	58/1
*Signal transduction mechanisms (3)*				
269	Calcium-activated outward-rectifying potassium channel, putative- *R*. *communis*	XP_002522760	5.24/38.87	65/1
293	BRASSINOSTEROID INSENSITIVE 1-associated receptor kinase 1 precursor, putative- *R*. *communis*	XP_002510316	8.48/80.56	50/1
365	auxin-induced protein 15A-like—*V*. *vinifera*	XP_002276347	7.76/10.63	54/1
*DNA binding protein (1)*				
176	DNA binding protein, putative—*R*. *communis*	XP_002532949	8.48/54.90	35/1
*Function unknown proteins (18)*				
205	hypothetical protein RCOM_0819620—*R*. *communis*	XP_002525182	9.50/84.66	48/1
227	conserved hypothetical protein—*R*. *communis*	XP_002530467	9.22/22.39	75/1
228	conserved hypothetical protein- *R*. *communis*	XP_002524093	9.52/52.59	55/1
261	hypothetical protein—*V*. *vinifera*	XP_002278636	6.18/35.52	95/1
263	Uncharacterized protein TCM_019766 -*T*. *cacao*	XP_007033598	8.77/9.87	72/1
264	predicted protein- *P*. *trichocarpa*	XP_002312583	6.11/35.72	324/2
278	predicted protein—*P*. *trichocarpa*	XP_002312583	6.11/35.72	342/2
294	conserved hypothetical protein—*R*. *communis*	XP_002526882	9.24/9.48	48/1
296	conserved hypothetical protein- *R*. *communis*	XP_002522170	9.01/49.07	56/1
298	conserved hypothetical protein—*R*. *communis*	XP_002528936	9.47/25.96	70/1
307	hypothetical protein RCOM_1516730—*R*. *communis*	XP_002524005	5.59/57.56	66/1
318	hypothetical protein RCOM_1516730—*R*. *communis*	XP_002524005	5.59/57.56	66/1
320	hypothetical protein RCOM_0908960—*R*. *communis*	XP_002517012	5.76/116.97	55/1
326	conserved hypothetical protein—*R*. *communis*	XP_002520953	8.52/22.91	55/1
328	conserved hypothetical protein—*R*. *communis*	XP_002521472	5.62/39.20	68/1
349	unknown—*Astragalus membranaceus*	AAW80931	5.77/19.69	51/1
367	hypothetical protein CICLE_v10008279mg—*Citrus clementina*	XP_006453534	7.93/43.56	52/1
368	conserved hypothetical protein—*R*. *communis*	XP_002520670	6.83/9.44	65/1
*SC205 (9)*				
*Carbohydrate and energy metabolism associated proteins (2)*				
408	NAD-malate dehydrogenase—*Nicotiana tabacum*	CAB45387	8.03/43.31	51/1
420	electron transfer flavoprotein-ubiquinone oxidoreductase, putative—*R*. *communis*	XP_002522858	5.44/37.89	68/1
*Detoxifying and antioxidant (2)*				
413	glutathione-s-transferase theta, gst, putative—*R*. *communis*	XP_002530205	6.24/24.87	88/1
430	ferritin, plant, putative—*R*. *communis*	XP_002526668	5.25/28.42	90/1
*Chaperones (1)*				
445	heat-shock protein, putative—*R*. *communis*	XP_002519929	6.21/22.19	90/1
*Amino acid metabolism (1)*				
418	phosphatidylserine decarboxylase, putative—*R*. *communis*	XP_002526445	5.96/71.01	57/1
*DNA and RNA metabolism associated proteins (1)*				
424	mta/sah nucleosidase, putative—*R*. *communis*	XP_002520036	5.06/29.31	63/1
*Function unknown proteins (2)*				
383	hypothetical protein POPTR_0002s26090g—*P*. *trichocarpa*	XP_006386918	4.93/26.04	65/1
398	hypothetical protein RCOM_1272620—*R*. *communis*	XP_002523685	8.73/38.15	55/1
*SC8 (12)*				
*Carbohydrate and energy metabolism associated proteins (5)*				
465	Transaldolase, putative—*R*. *communis*	XP_002512678	5.50/47.81	75/1
466	methionine sulfoxide reductase family protein—*P*. *trichocarpa*	XP_002318692	6.59/21.38	80/1
467	abhydrolase domain containing, putative—*R*. *communis*	XP_002521575	9.30/35.30	64/1
469	Glycogen synthase kinase-3 beta, putative—*R*. *communis*	XP_002515218	8.59/46.22	77/1
471	electron transfer flavoprotein-ubiquinone oxidoreductase, putative—*R*. *communis*	XP_002522858	5.44/37.89	105/1
*Detoxifying and antioxidant (3)*				
472	ascorbate peroxidase APX2*—M*. *esculenta*	AAX84679	5.31/27.67	410/3
474	glutathione s-transferase, putative—*R*. *communis*	XP_002532823	5.40/25.42	89/1
475	dehydroascorbate reductase, putative—*R*. *communis*	XP_002523030	5.78/23.56	95/1
*Protein biosynthesis (1)*				
468	ccaat-binding transcription factor subunit A, putative—*R*. *communis*	XP_002516901	6.63/18.00	88/1
*Function unknown proteins (3)*				
470	hypothetical protein POPTR_0011s11090g—*P*. *trichocarpa*	XP_002317457	5.46/72.39	54/1
473	hypothetical protein POPTR_0017s02700g—*P*. *trichocarpa*	XP_006372549	7.02/15.38	74/1
476	conserved hypothetical protein—*R*. *communis*	XP_002531502	8.46/6.24	60/1
*SC205 and SC8 (87)*				
*Carbohydrate and energy metabolism associated proteins (21)*				
369	aconitase—*Noteroclada confluens*	BAA06108	5.74/98.00	55/1
370	NADH-ubiquinone oxidoreductase, putative- *R*. *communis*	XP_002531931	6.56/80.77	322/3
375	Xyloglucan endotransglucosylase/hydrolase protein 22 precursor, putative- *R*. *communis*	XP_002526228	4.89/31.87	66/1
376	d-3-phosphoglycerate dehydrogenase, putative—*R*. *communis*	XP_002518687	7.65/63.10	421/4
385	ATP synthase subunit beta, mitochondrial	P17614	5.95/59.86	342/2
386	Enolase 2	Q9LEI9	5.92/47.91	75/1
389	sinapyl alcohol dehydrogenase—*Populus tremuloides*	AAK58693	6.23/38.99	67/1
394	ADPglucose pyrophosphorylase—*Oryza sativa*	AAA33891	5.65/53.51	117/2
395	ATP synthase subunit beta, mitochondrial; Flags: Precursor	P17614	5.95/59.86	643/4
400	2,3-bisphosphoglycerate-independent phosphoglycerate mutase	P35494	5.98/61.07	77/1
401	pyruvate dehydrogenase E1 beta subunit isoform 1- *Zea mays*	AAC72192	5.54/39.81	51/1
403	uroporphyrinogen decarboxylase, putative—*R*. *communis*	XP_002520842	6.68/42.84	60/1
406	sinapyl alcohol dehydrogenase—*P*. *tremuloides*	AAK58693	6.23/38.99	52/1
407	sinapyl alcohol dehydrogenase—*P*. *tremuloides*	AAK58693	6.23/38.99	53/1
414	NADP-dependent malic enzyme—*Camellia sinensis*	ACJ38230	7.92/26.71	44/1
425	Pectinesterase inhibitor, putative—*R*. *communis*	XP_002519258	8.69/19.28	62/1
433	triosephosphate isomerase—*Glycine max*	AAT46998	5.87/27.23	88/1
434	triosephosphate isomerase—*G*. *max*	AAT46998	5.87/27.23	95/1
435	dolichyl-phosphate mannose synthase- *Rothia dentocariosa ATCC 17931*	ZP_06905560	9.02/27.84	45/1
441	ATP binding protein, putative—*R*. *communis*	XP_002512849	7.53/35.20	88/1
460	N-acetyltransferase, putative—*R*. *communis*	XP_002517113	6.74/20.94	66/1
*Detoxifying and antioxidant (11)*				
377	NADP-dependent malic enzyme—*C*. *sinensis*	ACJ38230	7.92/26.71	84/1
397	aldo/keto reductase AKR- *Manihot esculenta*	AAX84672	6.38/37.71	386/4
402	isoflavone reductase homolog Bet v 6.0101—*B*. *pendula*	AAC05116	3.12/33.75	44/1
412	aldo/keto reductase AKR—*M*. *esculenta*	AAX84672	6.38/37.71	243/2
415	ascorbate peroxidase APX2—*M*. *esculenta*	AAX84679	5.31/27.67	571/5
416	L-ascorbate peroxidase- *P*. *sativum*	CAA43992	5.52/27.19	71/1
417	ascorbate peroxidase APX2- *M*. *esculenta*	AAX84679	5.31/27.67	423/3
419	stromal ascorbate peroxidase- *Spinacia oleracea*	BAA12039	8.46/39.52	57/1
428	ferritin, plant, putative—*R*. *communis*	XP_002526668	5.25/28.42	82/1
429	ferritin 2 precursor family protein—*P*. *trichocarpa*	XP_002315139	6.21/22.19	80/1
456	copper/zinc superoxide dismutase—*M*. *esculenta*	AAT77951	5.42/15.11	90/1
*Chaperones (10)*				
371	HSP68	AAB26551	5.20/62.38	142/2
373	chaperonin precursor—*Pisum sativum*	AAA66365	5.85/62.98	160/3
379	rubisco subunit binding-protein alpha subunit, ruba, putative—*R*. *communis*	EEF28034	5.25/53.20	142/4
427	ubiquitin-protein ligase, putative—*R*. *communis*	XP_002525340	7.44/21.80	58/1
436	small heat-shock protein, putative-*R*. *communis*	XP_002517628	7.79/26.74	108/1
444	Ran GTPase binding protein, putative—*R*. *communis*	XP_002519871	5.68/37.55	92/1
446	heat-shock protein, putative—*R*. *communis*	XP_002519929	6.21/22.19	86/1
449	heat-shock protein, putative—*R*. *communis*	XP_002530396	6.34/18.42	123/1
451	heat-shock protein, putative- *R*. *communis*	XP_002526950	5.81/15.36	123/1
459	17.5 kDa class I heat shock protein	P04793	5.33/17.55	76/1
*Structure (6)*				
392	actin 1—*Boehmeria nivea*	ABG49457	5.31/41.67	359/4
393	actin family protein—*P*. *trichocarpa*	XP_002298710	5.31/41.70	78/1
437	annexin, putative—*R*. *communis*	XP_002513082	5.88/36.02	80/1
442	Centromeric protein E, putative—*R*. *communis*	XP_002509929	4.76/197.60	57/1
455	Charged multivesicular body protein 2a, putative—*R*. *communis*	XP_002521345	5.82/25.36	88/1
458	Major latex protein, putative—*R*. *communis*	XP_002534267	5.43/16.83	67/1
*Protein biosynthesis (5)*				
372	acyl-peptide hydrolase-like—*A*. *thaliana*	BAB09360	5.08/75.42	70/1
380	protein disulphide isomerase PDI—*R*. *communis*	AAB05641	4.95/55.56	40/1
384	Protein disulfide-isomerase	Q43116	4.95/55.56	193/2
426	Proteasome subunit alpha type-6	O48551	5.83/27.39	281/1
431	proteasome subunit beta type 6,9, putative—*R*. *communis*	XP_002527995	5.17/24.87	219/3
*Photosynthesis related proteins (2)*				
388	Ribulose bisphosphate carboxylase large chain	P28427	6.60/51.81	436/3
464	photosystem I subunit VII—*M*. *esculenta*	YP_001718487	6.68/9.05	98/1
*DNA and RNA metabolism associated proteins (2)*				
457	Nucleoside diphosphate kinase B	P47920	6.42/16.20	63/1
461	glycine-rich RNA-binding protein—*Citrus unshiu*	BAA92156	7.85/16.85	135/2
*Amino acid metabolism (1)*				
405	S-adenosylmethionine synthase 2	P17562	5.67/43.26	68/1
*HCN metabolism (1)*				
374	Linamarase—*M*. *esculenta*	AAB22162	5.52/61.37	104/1
*Transport (1)*				
409	Thiazole biosynthetic enzyme, chloroplastic	Q38709	5.42/37.06	109/1
*Defense (1)*				
396	putative NBS-LRR disease resistance protein—*Malus floribunda*	ABG24002	8.35/17.63	70/2
*Inorganic ion transport and metabolism (2)*				
439	Calmodulin, putative- *R*. *communis*	XP_002514520	4.16/17.49	
452	Calmodulin—*A*. *thaliana*	CAA78058	4.20/15.60	95/2
*DNA binding protein (2)*				
391	Nucleotide binding, putative—*T*. *cacao*	XP_007040180	9.20/9.78	62/1
447	DNA binding protein, putative—*R*. *communis*	XP_002516111	5.10/28.52	70/1
*Function unknown proteins (22)*				
378	hypothetical protein RCOM_0537780—*R*. *communis*	XP_002530006	4.64/41.36	106/1
381	hypothetical protein ARALYDRAFT_492381—*Arabidopsis lyrata subsp*. *lyrata*	EFH43914	5.42/67.13	35/1
382	conserved hypothetical protein—*R*. *communis*	XP_002519078	6.71/27.52	74/1
387	hypothetical protein—*V*. *vinifera*	XP_002283022	6.38/63.80	239/2
390	hypothetical protein—*V*. *vinifera*	XP_002274975	10.42/44.77	313/3
399	conserved hypothetical protein—*R*. *communis*	XP_002525028	8.45/7.91	60/1
404	protein with unknown function—*R*. *communis*	XP_002519452	6.00/23.68	75/1
410	predicted protein—*P*. *trichocarpa*	XP_002312583	6.11/35.72	347/2
411	unnamed protein product—*A*. *thaliana*	BAB09870	7.69/91.25	48/1
421	hypothetical protein RCOM_0722880—*R*. *communis*	XP_002524850	6.29/45.50	70/1
422	conserved hypothetical protein—*R*. *communis*	XP_002519888	10.02/7.63	57/1
423	conserved hypothetical protein—*R*. *communis*	XP_002523631	8.14/31.96	65/1
432	unnamed protein product—*G*. *max*	CAN08825	5.16/25.13	50/1
438	Major allergen Pru ar, putative- *R*. *communis*	XP_002516987	4.68/17.65	66/1
440	hypothetical protein PRUPE_ppa023969mg, partial—*Prunus persica*	XP_007199337	7.85/17.25	72/1
443	conserved hypothetical protein—*R*. *communis*	XP_002515887	10.33/10.83	54/1
448	predicted protein—*P*. *trichocarpa*	XP_002329730	6.74/17.45	135/1
450	conserved hypothetical protein—*R*. *communis*	XP_002523034	4.80/11.55	71/1
453	hypothetical protein CICLE_v10023197mg—*Citrus clementina*	XP_006439559	9.42/7.87	56/1
454	conserved hypothetical protein—*R*. *communis*	XP_002537812	8.10/7.89	60/1
462	conserved hypothetical protein—*R*. *communis*	XP_002534082	5.60/18.28	98/1
463	predicted protein—*P*. *trichocarpa*	XP_002324923	5.74/10.93	129/1
*SC205 and W14 (5)*				
*Carbohydrate and energy metabolism associated proteins (2)*				
234	Enolase 2	Q9LEI9	5.92/47.91	151/1
244	sinapyl alcohol dehydrogenase—*P*. *tremuloides*	AAK58693	6.23/38.99	53/1
*Structure (1)*				
358	Major latex protein, putative—*R*. *communis*	XP_002534267	5.43/16.83	66/1
*Protein biosynthesis (1)*				
203	26S proteasome non-atpase regulatory subunit, putative- *R*. *communis*	XP_002529379	4.71/27.99	70/1
*Function unknown proteins (1)*				
351	hypothetical protein RCOM_0494170—*R*. *communis*	XP_002531249	5.99/36.25	61/1
*SC8 and W14 (6)*				
*Carbohydrate and energy metabolism associated proteins (1)*				
222	ATP synthase subunit beta vacuolar, putative—*R*. *communis*	XP_002510596	4.99/54.35	90/1
*Amino acid metabolism (2)*				
310	remorin—*M*. *indica*	AGB07445	5.69/22.02	80/1
360	Major latex protein, putative—*R*. *communis*	XP_002534267	5.43/16.83	75/1
*Function unknown proteins (3)*				
268	hypothetical protein 30—*H*. *brasiliensis*	ADR71308	5.59/11.93	55/1
334	conserved hypothetical protein—*R*. *communis*	XP_002534004	9.72/18.16	70/1
355	predicted protein—*P*. *trichocarpa*	XP_002329730	6.74/17.45	135/1
The total protein number			177	

^a^, The numbers corresponded to the 2-DE gels in Fig 6 and S5 Fig;

^b^, NCBI accession number;

^c^, Probability-based MOWSE (molecular weight search) scores;

^d^, The number of unique peptides identified by MS/MS, and individual ions scores are all identity or extensive homology (p<0.05).

### Functional classification of identified proteins

**Fig 8:**
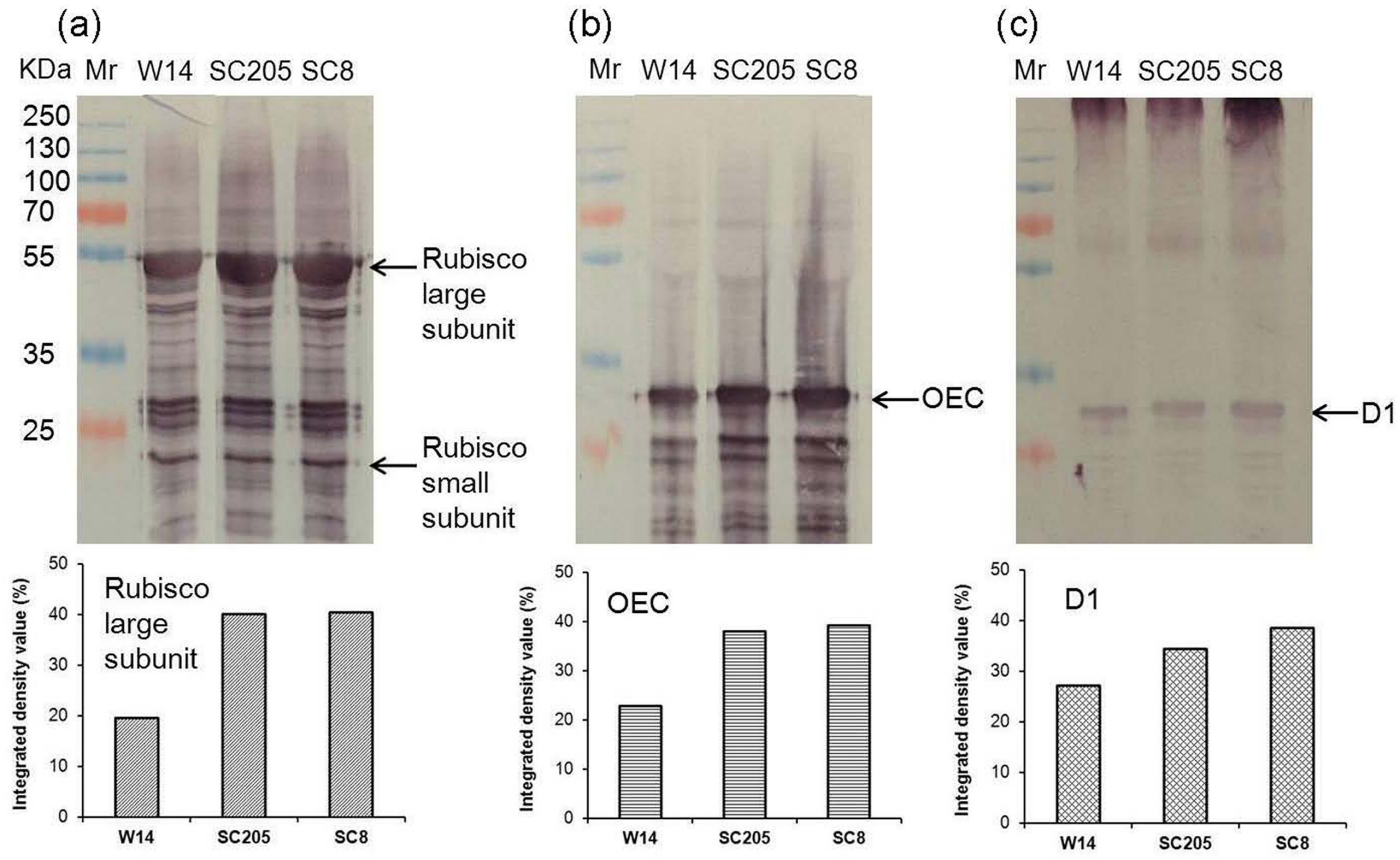
Western blotting of Rubisco (a), OEC (b) and D1 (c). The expression of Rubisco, OEC and D1 in leaves of cassava W14, SC205 and SC8 were detected by western blotting using anti-Rubisco-polyclonal antibody (AS07218), anti-OEC antibody (AS 05092) and anti-D1 antibody (AS05084) from Agrisera, respectively.

Of the identified proteins, 175 proteins in leaves were annotated via the survey of gene banks (Fig 5a and 5b, Tables 3 and 4). These proteins were associated with photosynthesis (22.3%), carbohydrate and energy metabolism (24.0%), detoxifying and antioxidants (7.4%), defense (4.6%), protein biosynthesis (8.0%), chaperones (6.3%), HCN metabolism (3.4%), structure (4.0%), amino acid metabolism (3.4%), signal transduction mechanisms (1.7%), inorganic ion transport and metabolism (0.6%), DNA binding proteins (0.6%) and proteins of unknown function (13.7%). Twenty differential proteins including common proteins of W14, SC205 and SC8 (spots, 18, 32, 36, 37, 38, 40, 60, 62, 105, 113), W14 unique proteins (spot 46), SC8 unique proteins (spots, 174, 175), SC205 and SC8 common proteins (spots, 152, 166, 168, 169, 171, 172) and SC205 and W14 common proteins (spot 63) were associated with Rubisco proteins (Tables 3 and 4). Of these, 8 up-regulated proteins (spots, 18, 32, 36, 37, 38, 40, 62, 105) in the pairwise comparison of SC205/W14 and SC8/W14 were detected. Immunoblotting results showed that Rubisco expression in SC205 and SC8 was higher than that in W14 (Fig 8a), which was similar with the 2-DE result. The expressed levels of proteins OEC and D1 related with photosynthesis in SC205 and SC8 were higher than that in W14 (Fig 8b and 8c).

**Fig 9:**
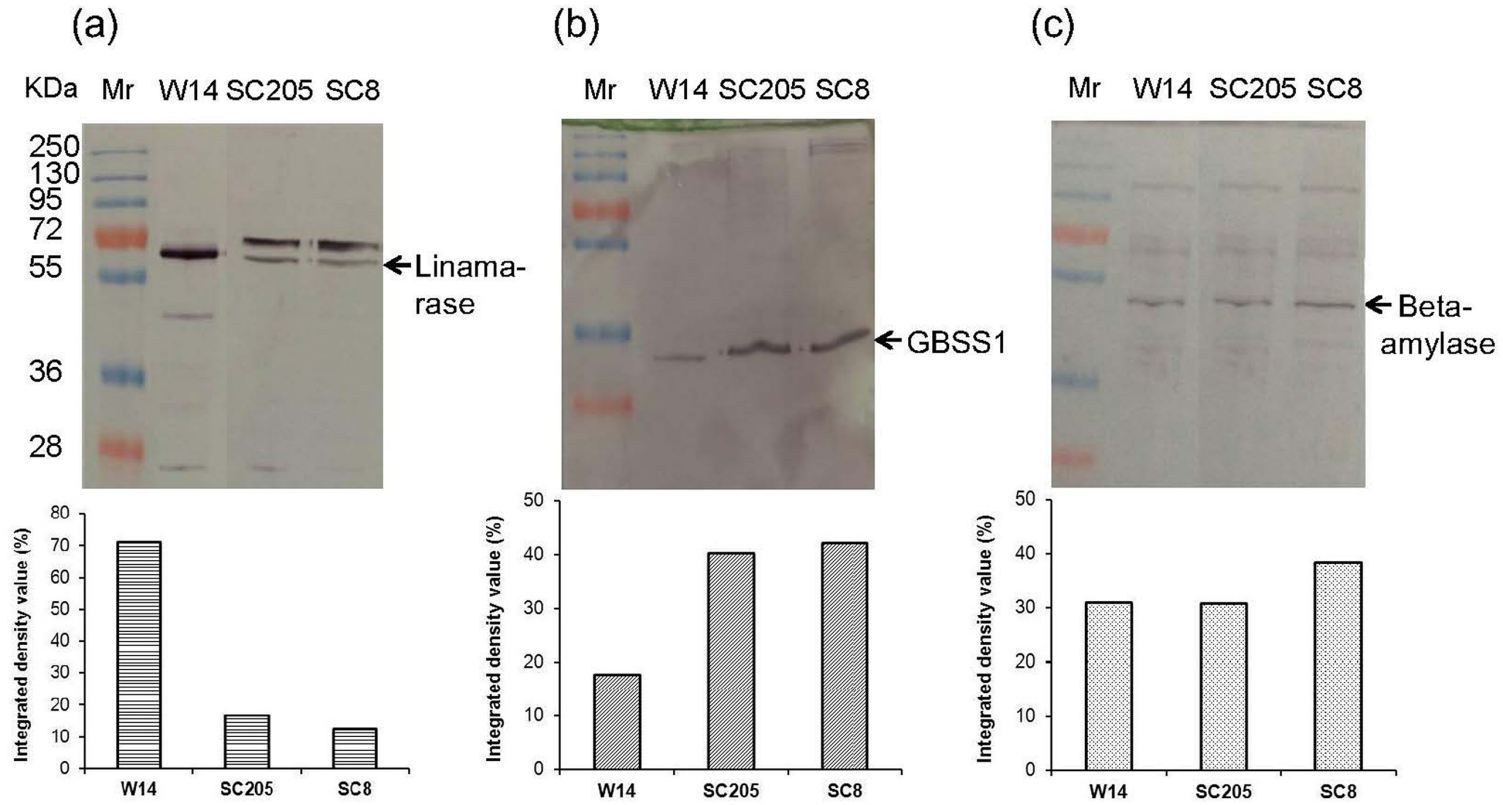
Western blotting of linamarase (a), GBSS1 (b) and beta-amylase (c). The expression of linamarase, GBSS1and beta-amylase in storage root of cassava W14, SC205 and SC8 genotypes were detected by western blotting using anti- linamarase antibody anti- GBSS1 antibody, produced by GenScript, and anti-beta-amylase antibody (AS09379) from Agrisera.

A total of 304 identified proteins in storage roots were annotated according to gene ontology (Fig 7a and 7b, Tables 5 and 6). These proteins were related with carbohydrate and energy metabolism (28.3%), chaperones (11.5%), detoxifying and antioxidant (9.5%), structure (6.3%), amino acid metabolism (3.3%), protein biosynthesis (6.9%), photosynthesis (1.3%), DNA and RNA metabolism (3.3%), defense (1.3%), HCN metabolism (1.0%), signal transduction (2.3%), transport (1.6%) and proteins of unknown function (21.7%). Of those, three protein spots (spots 191, 209, 374) were detected to be linamarase proteins, associated with HCN mechanism (Tables 5 and 6). Expression of linamarase in storage roots of W14, SC205 and SC8 were confirmed with immunoblotting (Fig 9a). The linamarase expression in SC205 and SC8 was less than that in W14. The western blot results also showed that the expression of GBSS1 in W14, regulated the amylose synthesis, was less than that in SC205 and SC8 (Fig 9b). However, the beta-amylase expression in SC8 was slightly higher than that in SC205 and W14 (Fig 9c).

### Protein interaction networks

**Fig 10:**
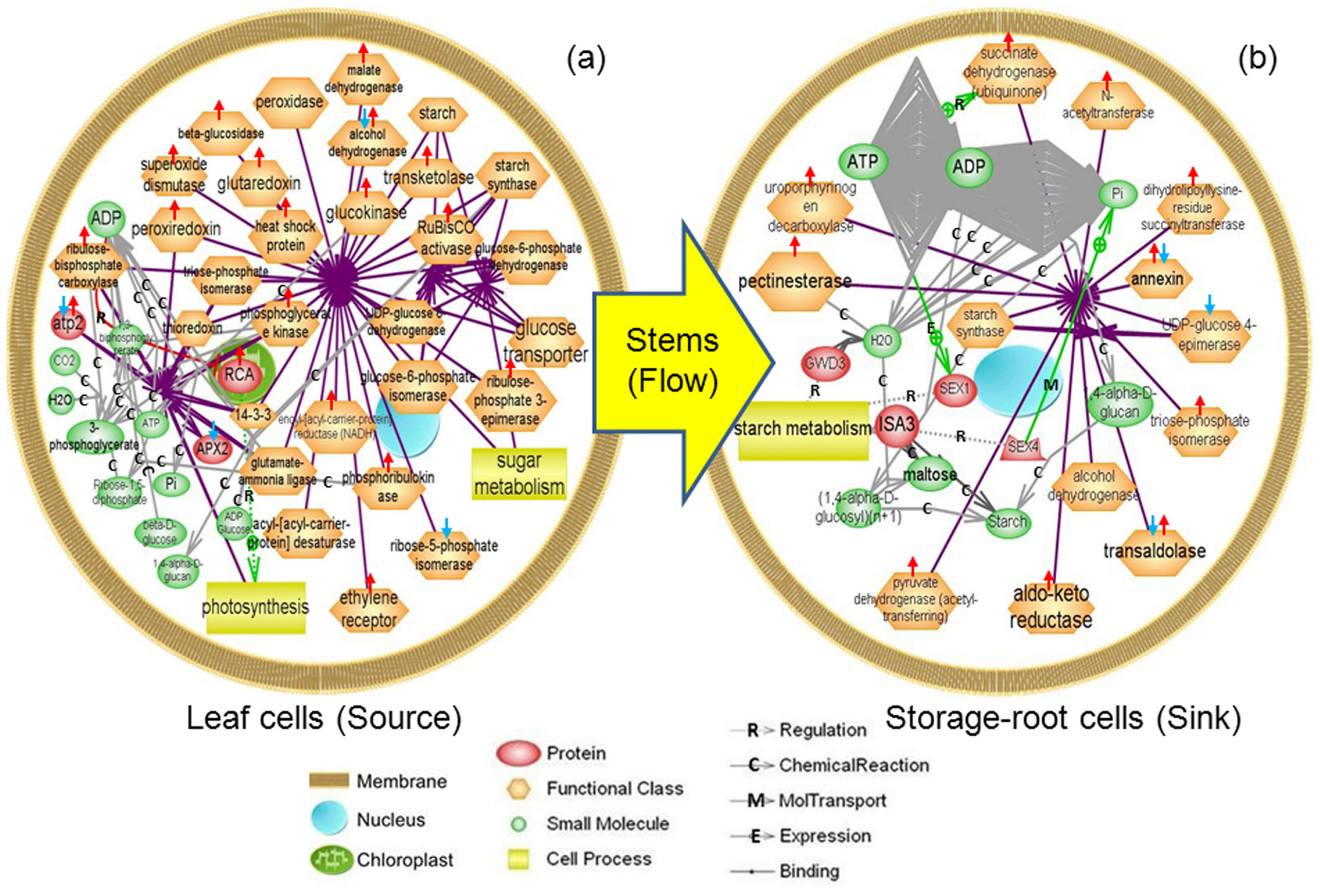
Biological networks generated from a combination of 19 differential proteins involved with photosynthesis (a) in cassava leaves and 11 differential proteins related with starch accumulation (b) in storage roots. Nineteen differentially up-(a red and upward arrow) and down-(a blue and downward arrow) regulated proteins including ribulose-bisphosphate carboxylase, phosphoribulokinase, ribulose- phosphate-3-epimerase, ribose-5-phosphate isomerase, RCA, transketolase, ATP synthase subunit beta, phosphoglycerate kinase, malate dehydrogenase, alcohol dehydrogenase and enoyl-ACP reductase, ethylene receptor, peroxiredoxin, heat shock protein, glucokinase, glutaredoxin, superoxide dismutase, beta-glucosidase and APX2 in cassava cultivars were used to generate a protein-protein interaction network about photosynthesis through Pathway Studio analysis. Eleven differentially up-(a red and upward arrow) and down-(a blue and downward arrow) regulated proteins including succinate dehydrogenase, dihydrolipoyllysine-residue succinyltransferase, UDP- glucosyltrans-ferase, transaldolase, uroporphyrinogen decarboxylase, pectinesterase, triosephosphate isomerase, N-acetyltransferase, aldo-keto reductase, annexin and pyruvate dehydrogenase in cassava cultivars were used to generate a protein-protein interaction network regarding starch accumulation through Pathway Studio analysis. Regulation is marked as an arrow with R, Chemical Reaction as an arrow with C, MolTransport as an arrow with M, Expression as an arrow with E and Binding as an arrow without any marks. The entity table and relation table were presented in S1 and S2 Tables.

A protein interaction map was generated with 19 differential proteins involved with photosynthesis (Fig 10a) and 11 differential proteins related with starch accumulation (Fig 10b). The interactional relationships between the 30 differential proteins included regulation, chemical reaction, molecular transport, expression and binding, responding to photosynthesis, sugar metabolism, and starch metabolism (S1 and S2 Tables). There were direct interactions between 17 up- and 4 down-regulated proteins, associated with photosynthesis and sugar metabolism, in SC205 and SC8 compared with W14 (Fig 10a). Of these, ribulose-bisphosphate carboxylase, phosphoribulokinase, ribulose-phosphate-3-epimerase, ribose-5-phosphate isomerase, RCA, transketolase, ATP synthase subunit beta, phosphoglycerate kinase, malate dehydrogenase, alcohol dehydrogenase and enoyl-ACP reductase would be directly involved with photosynthesis and carbohydrate and energy metabolism (Tables 3 and 4). The analysis of differential proteins in storage roots showed that there were direct interactions between 10 up- and 3 down-regulated proteins involved in starch accumulation in SC205 and SC8 compared with W14 (Fig 10b), in which succinate dehydrogenase, dihydrolipoyllysine-residue succinyltransferase, UDP-glucosyltrans-ferase, transaldolase, uroporphyrinogen decarboxylase, pectinesterase, triosephosphate isomerase, N-acetyltransferase were related with carbohydrate and energy metabolism (S1 and S2 Tables).

## Discussion

In the present study it is the first time to investigate the differences of anatomy and physiology associated with leaf photosynthesis and starch accumulation of storage roots in combination with proteomic technique between cassava cultivars and the wild relative. In this study, we also focused on understanding the relation between the pathways of photosynthesis and starch accumulation, and then indicated the regulated mechanism from photosynthesis to starch synthesis demonstrated by the phenotype of W14, which is a cassava wild species with low starch content.

### Proteome changes in photosynthetic activity in leaves

*M*. *esculenta* ssp. *Flabellifolia*, the potential wild progenitor of *M*. *esculenta*, exhibits typical traits of C_3_ photosynthesis, indicating that cultivated cassava, despite its peculiar photosynthetic characteristics, is not derived from wild C_4_ species [22]. The mesophyll surrounds the bundle sheath cells, where CO_2_ is enriched around Rubisco and the reduction of carbon takes place. The chloroplasts of mesophyll and bundle sheath tissues are adapted to their respective roles [23]. Li *et al*. (2010) identified 110 proteins from plantlet leaves of cassava genotype SC8 using LC-ESI-MS/MS, of these, the proteins involved in photosynthesis were among the largest group (21.8%). Photosynthetic enzymes are abundantly expressed in green tissues, in which Rubisco represents about 50% of the total protein content in leaves, and may be among the controlled keys of the photosynthetic pathways. Oxygen-evolving enhancer protein 1 (OEE1), are involved in photosynthesis and may be synthesized in the shoots and then transported to the roots [8]. In the C_3_ pathway, CO_2_ is fixed by Rubisco and is incorporated into carbohydrate. This metabolic pathway operates only in the mesophyll cells. Leaves of C_4_ plants display Kranz anatomy, in which vascular bundles are surrounded by an outer layer of the mesophyll cells and an inner layer of bundle sheath cells [24, 25]. As showed in Fig 1, cassava leaves have distinct green bundle-sheath cells, with small, thin-walled cells, spatially separated below the palisade cells (different from Karanz-type leaf anatomy)[26], suggesting cassava is intermediate between C_3_ and C_4_ species [27].

In the present study, 13 (11 up-regulated and 2 down-regulated) and 11 (10 up-regulated and 1 down-regulated) differentially expressed proteins are directly involved with photosynthesis metabolism in leaves by pairwise comparison of SC205/W14 and SC8/W14, respectively (Table 3). Nice unique proteins related with photosynthesis were also detected in cultivars compared with W14 (Table 4). Therefore, the cultivars may have a higher photosynthetic rate than its wild relative W14. The proteome data imply that up-regulated protein patterns may be related with the increased photosynthetic activities. In two cultivars, the expressions of 9 differential proteins associated with photosynthesis in SC8 were higher than that in SC205, indicating SC8 has higher photosynthetic activity than SC205 (Table 3). These results were consistent with the data provided from measurement of photosynthetic activities using Imaging PAM (Fig 2 and Table 1) and the leaf anatomy (Fig 1). The photosynthesis performance, the expression of C_3_ photosynthetic enzymes Rubisco (Fig 8a) and higher resource use efficiency indicate that *M*. *esculanta* is likely to be a C_3_ and C_4_ species. Relative to its wild relative *M*. *esculenta* ssp. *Flabellifolia*, the higher carboxylation efficiency and greater resource use efficiency of *M*. *esculenta* are due to its markedly higher C_3_ photosynthetic enzyme activities. The high expression of OEC and D1 related with photosynthesis detected by Western blot also supported the result described above (Fig 8b and 8c).

### Proteome changes in starch accumulation in storage roots

The cassava storage root, a vegetative structure, accumulates starch as a reserve compound [28] and has no reproductive properties such as for potato tubers. It develops from fibrous roots through massive cell division and differentiation of parenchyma cells of the secondary xylem [19]. However, not all fibrous roots are designated for storage root formation. Little is known about the mechanism involved in the transition from fibrous roots to storage roots. Li *et al*. (2010) identified 147 proteins present in cassava adventitious roots, and 155 proteins in storage roots of cassava genotype SC8. Of these, a total of 37 proteins were present in both adventitious and storage roots, 74 unique proteins to adventitious roots and 102 unique proteins to storage roots, indicating that the two types of roots have both overlapping and different metabolic activities [8].

Starch is the main form in which plants store carbon. In the present study, 10 (8 up-regulated and 2 down-regulated) and 9 (7 up-regulated and 2 down-regulated) differential expressed proteins were related with carbohydrate and energy metabolism in storage roots by pairwise comparison of SC205/W14 and SC8/W14, respectively (Table 5). Twenty one unique proteins associated with carbohydrate and energy metabolism were also found in cultivars compared with W14 (Table 6). These up-regulated proteins (AGPase, enolase, and aconitase) in cultivars are associated with starch synthesis, glycolysis and TCA cycle, implying cultivars have a higher starch accumulation than its wild relatives. Starch occurs as semi-crystalline granules composed of two polymers of glucose, called amylose and amylopectin. Starch granules are characterized by internal growth rings. There is enormous variation in granule size and shape between plant organs, and between species [19]. The western blot showed that GBSSI, a key enzyme of amylose synthesis, had higher expression in cultivars SC205 and SC8 more than that in the wild relative W14 (Fig 9b). These data were consistent with the measurement of starch and amylose content in the storage roots of SC205, SC8 and W14 (Table 2), indicating storage root enlargement will coincide with the strong regulation of proteins associated with starch biosynthesis. In addition, activation of ADP-glucose pyrophosphorylase (AGPase), a key enzyme in starch synthesis, resulted in a stimulation of starch synthesis and decreased levels of glycolytic intermediates [29]. In the present study we observed that the AGPase (spot 255) was 3.6 and 2.5-fold more highly expressed in storage roots by pairwise comparison of SC205/W14 and SC8/W14, respectively (Table 5). This result was supported by the starch content analysis and amount calculation of starch granules using light microscope between SC205, SC8 and W14 (Fig 3 and Table 2).

### Linkages of photosynthesis and storage roots

The photosynthetic rate of cassava is very high and photosynthesis has a broad temperature optimum ranging from 20°C to 45°C [30]. It is cultivated worldwide for the high yield of its storage root containing high amount of starch. However, while our understanding of what is considered their primary function, i.e. starch accumulation and high photosynthetic rate, has increased dramatically in the recent years, relatively little is known about metabolic changes mediated by leaf-root interactions. To help fill in this gap, the anatomic and physiological analysis in combination with proteomics and bioinformatics between cultivated cassava and its wild relative with a low starch content has been employed to indicate the changes of enzyme activities and the expression levels of global proteins and their linkages between photosynthesis and starch accumulation in the present study. Tables 1and 2 revealed that low photosynthetic activity of W14 leaves resulted in the low dry matter and starch contents in the storage roots compared with cassava cultivars. As shown in Fig 10a, the processes of photosynthesis, starch and sugar metabolism (glycolysis, TCA cycle and pentose phosphate pathway) in cassava leaves produced many intermediate products for starch synthesis, i.e. ADP glucose, 1,4-alpha-D-glucan and Pi (Fig 10a). There are direct interactions between 17 up- and 4 down-regulated proteins, associated with photosynthesis and sugar metabolism, in SC205 and SC8 compared with W14, creating a strong source in cultivated cassava was more than that in its wild relatives (S1 and S2 Tables). A similar association between starch accumulation and the changes of global proteins was also evident in storage roots. Ten up- and 3 down-regulated proteins were detected to involve with starch and sucrose metabolism in cultivated cassava SC205 and SC8 compared with W14, suggesting a strong sink in cultivated cassava was more than that in its wild relatives (Fig 10b and S1 and S2 Tables). In the present study the biological network was established to explain the metabolic changes mediated by leaf-root interactions between cultivated cassava and its wild relatives in global-protein levels. It indicated that the positive crosstalk between the pathways of photosynthesis and starch accumulation in cultivated cassava resulted in the increase of starch content more than that in its wild relatives (Table 2).

## Conclusions

Overall, this study has generated the first comprehensive cassava protein data to show the proteome differences in leave and storage roots between cultivated cassava and its wild relatives due to the genetic differentiation. We detected 148, 157 and 152 leaf-protein spots, as well as 196, 228 and 232 storage-root-protein spots from 2-DE gels of W14, SC205 and SC8, respectively. A total of 175 proteins in leaves and 304 proteins in storage roots were identified, and classified into 12 functional groups annotated via the survey of gene banks. We also developed a biological network to indicate that the positive crosstalk between photosynthesis and starch accumulation may result in the increase of starch content in the storage roots. This implied that both photosynthesis and starch accumulation are equally important for increasing yield of cassava storage roots. We suggested that the divergence in proteome between cultivated cassava and its wild relative was caused by their genetic differentiation and the different number of genes encoding the differential proteins. The detailed analysis in a comprehensive dataset of global proteins and the large-scale bioinformatics will provide a clue for understanding the mechanism of leaf-root interactions and be helpful to choose the key protein markers involved with high starch content in cassava breeding.

## Supporting Information

S1 Fig Plant type, leaf and storage root of cassava cultivated SC205, SC8 and wild species W14. (a1), (a2) and (a3), plant type, leaf shape and storage root of W14, respectively; (b1), (b2) and (b3), plant type, leaf shape and storage root of SC205, respectively; (c1), (c2) and (c3), plant type, leaf and storage root of SC8, respectively.
(TIFF)

S2 Fig 2D gel image showing 122 proteins common in SC205, SC8 and W14 leaves. (TIFF)

S3 Fig 2D gel image showing unique proteins in W14 (a), SC205 (b) and SC8 (c) leaves. (TIFF)

S4 Fig 2D gel image showing 127 proteins common in SC205, SC8 and W14 storage roots. (TIFF)

S5 Fig 2D gel showing unique proteins in W14 (a), SC205 (b) and SC8 (c) storage roots. (TIFF)

S1 Table Entity table views of protein-protein interactions in biological networks generated for cassava photosynthesis (a) and starch accumulation (b). (XLS)

S2 Table Relation table views of protein-protein interactions in biological networks generated for cassava photosynthesis (a) and starch accumulation (b). (XLS)
