# Supplementary figures and images for: Domestication Syndrome Is Investigated by Proteomic Analysis between Cultivated Cassava (Manihot esculenta Crantz) and Its Wild Relatives
Source: PLoS One. 2016 Mar 29;11(3):e0152154. doi: 10.1371/journal.pone.0152154 (PMC4811587; doi:10.1371/journal.pone.0152154)

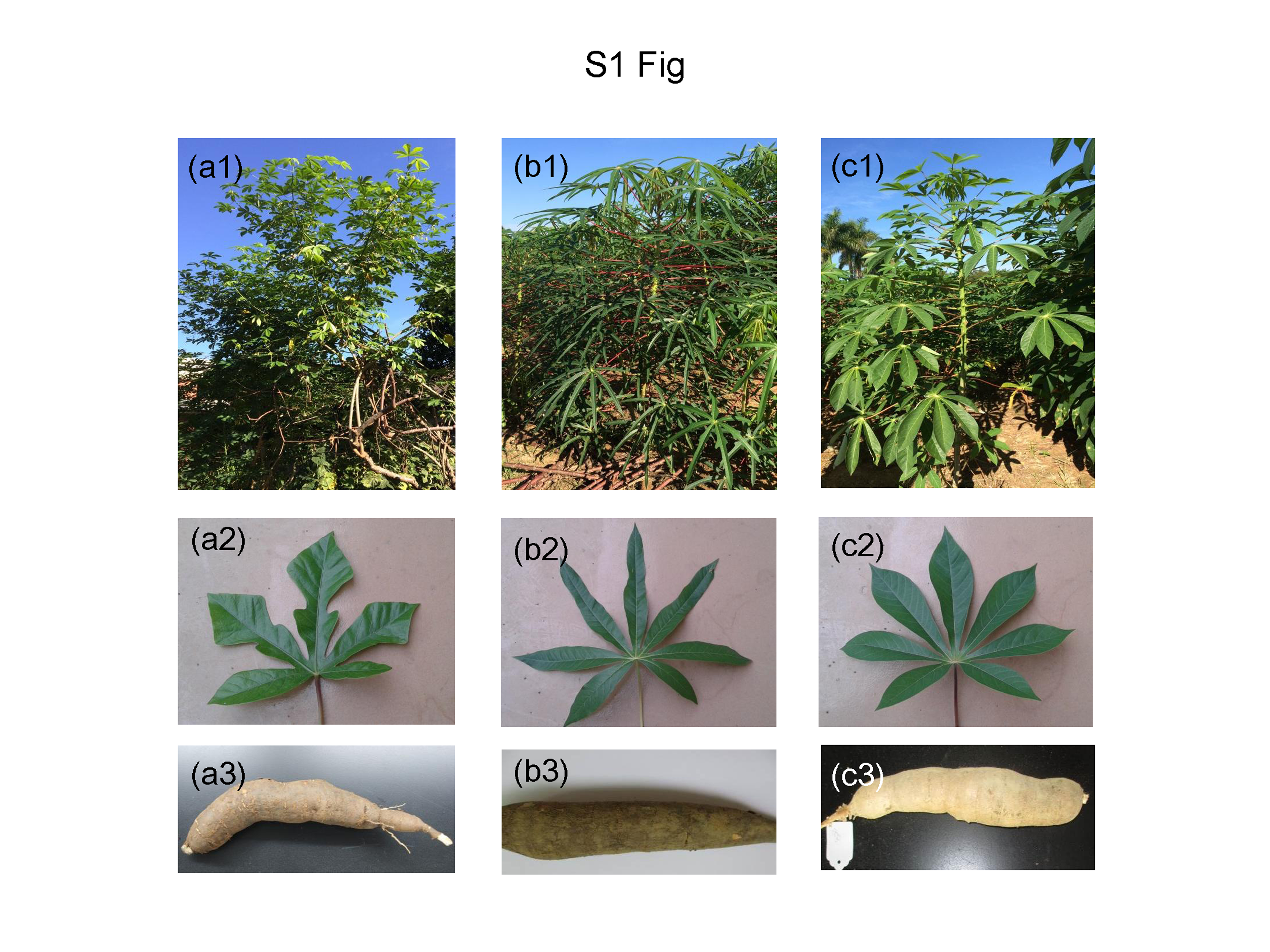

Supplement: S1 Fig — (a1), (a2) and (a3), plant type, leaf shape and storage root of W14, respectively; (b1), (b2) and (b3), plant type, leaf shape and storage root of SC205, respectively; (c1), (c2) and (c3), plant type, leaf and storage root of SC8, respectively. (TIFF) [file pone.0152154.s001.tiff]

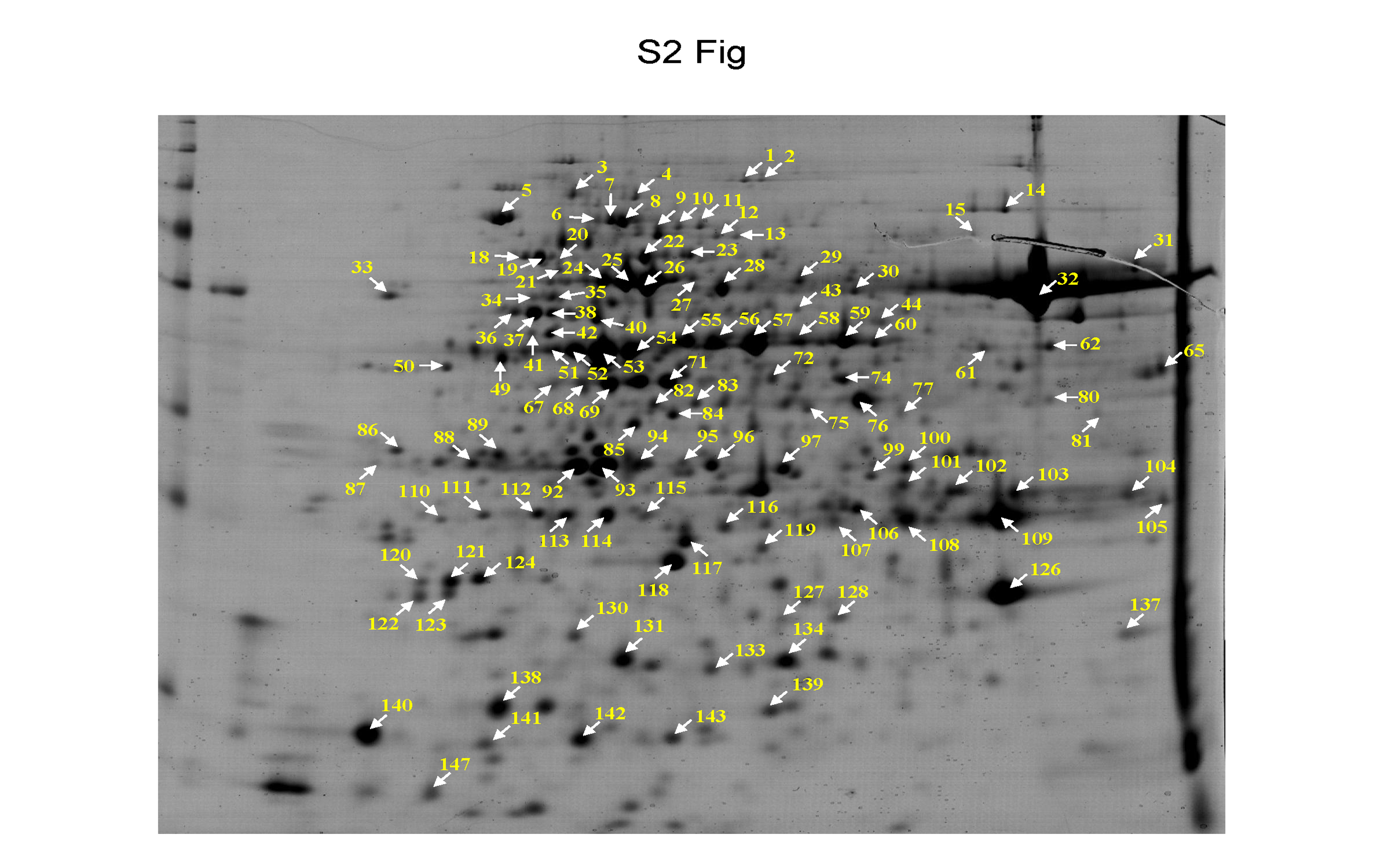

Supplement: S2 Fig — (TIFF) [file pone.0152154.s002.tiff]

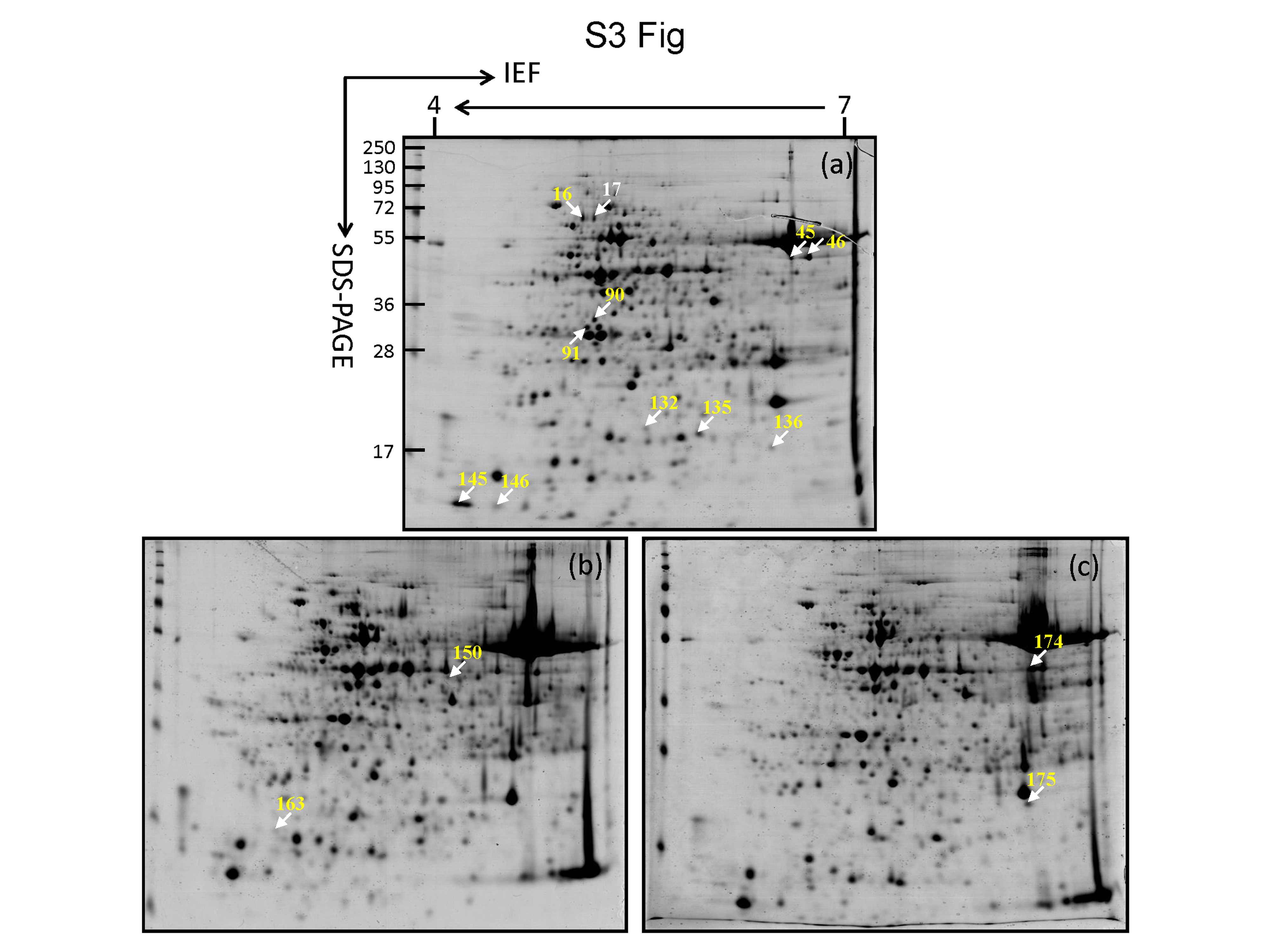

Supplement: S3 Fig — (TIFF) [file pone.0152154.s003.tiff]

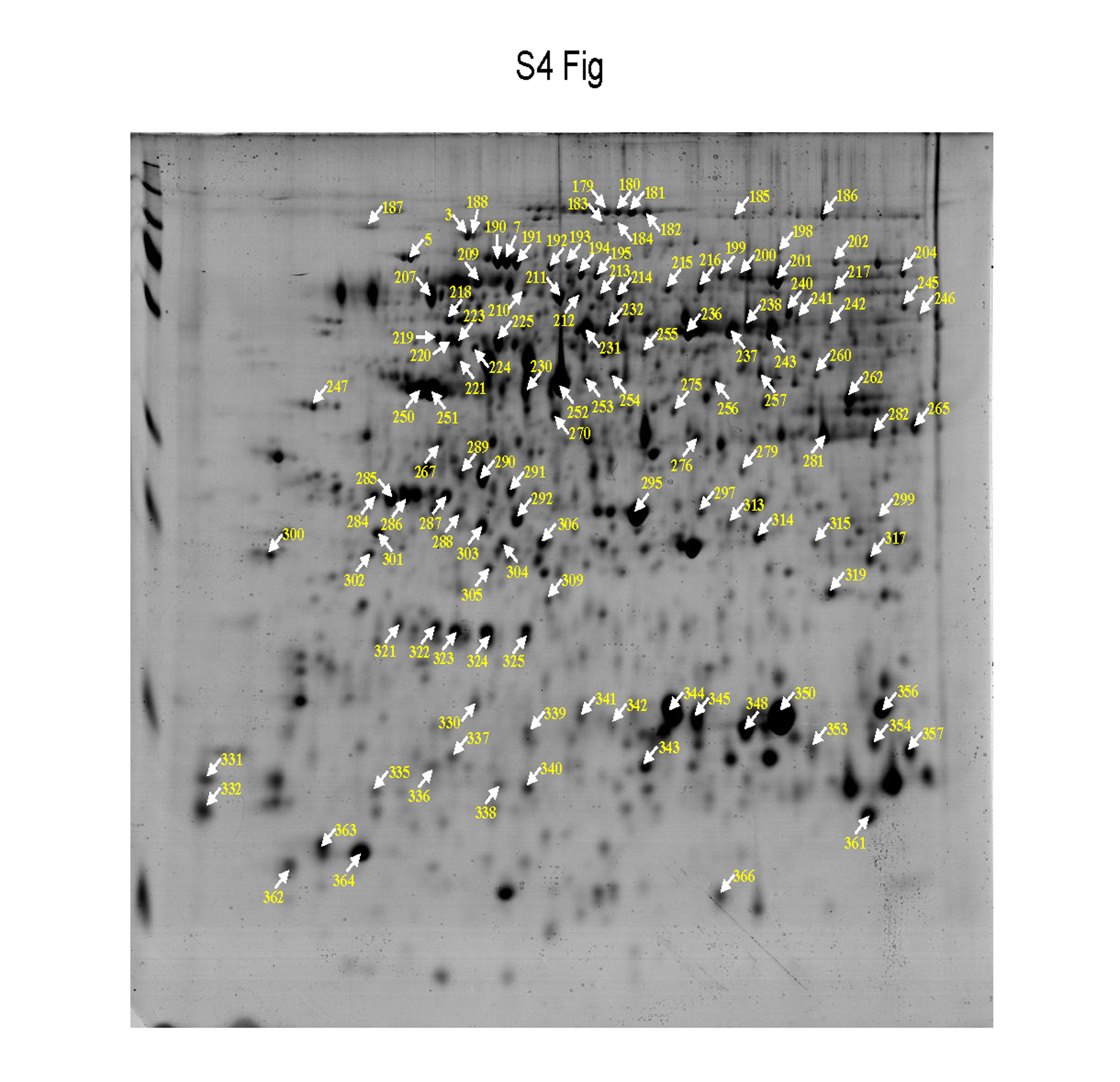

Supplement: S4 Fig — (TIFF) [file pone.0152154.s004.tiff]

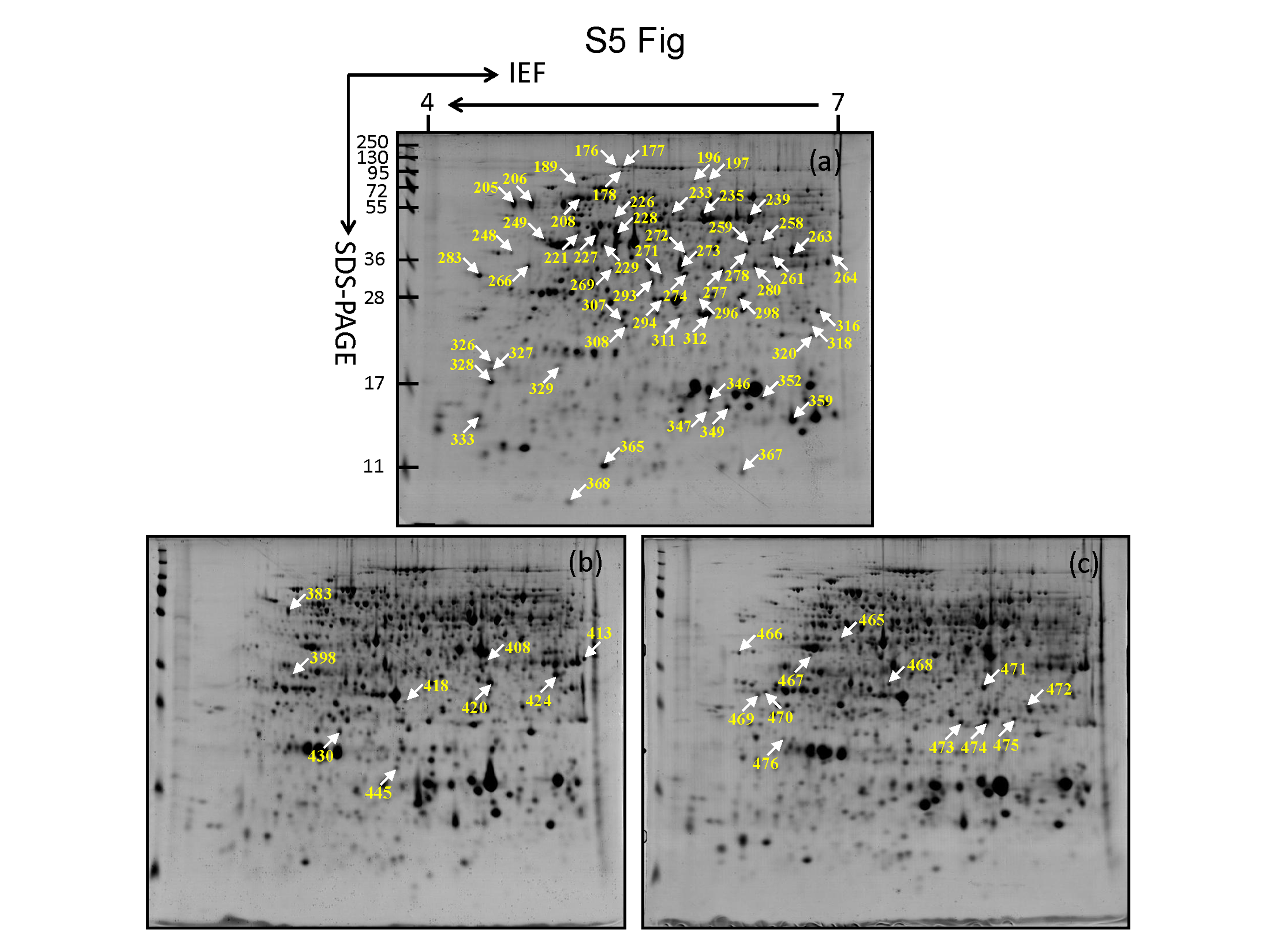

Supplement: S5 Fig — (TIFF) [file pone.0152154.s005.tiff]
